# Supplementary material for: Exploring post acute rehabilitation service use and outcomes for working age stroke survivors (≤65 years) in Australia, UK and South East Asia: data from the international AVERT trial
Source: BMJ Open. 2020 Jun 11;10(6):e035850. doi: 10.1136/bmjopen-2019-035850 (PMC7295421; doi:10.1136/bmjopen-2019-035850)
Supplement: Supplementary data [file bmjopen-2019-035850supp001.pdf]

Supplement 1

S-Table 1 Proportion of stroke survivors using different rehabilitation services in each region by two major prognostic factors: stroke severity and age

Region	Stroke Severity & Age	n	Inpatient Rehabilitation & Community Rehabilitation	Inpatient Rehabilitation & No Community Rehabilitation	Home & Community Rehabilitation	Home & No Rehabilitation Service Use
			**n=150**	**n=38**	**n=71**	**n=89**
**AUS**	Mild	209	59 (28%)	12 (6%)	58 (28%)	80 (38%)
	Mod	103	67 (65%)	15 (15%)	12 (12%)	9 (9%)
	Severe	36	24 (67%)	11 (31%)	1 (3%)	0 (0%)
	18-45	54	23 (43%)	1 (2%)	14 (26%)	16 (30%)
	46-65	294	127 (43%)	37 (13%)	57 (19%)	73 (25%)
			**n=26**	**n=12**	**n=78**	**n=35**
**UK**	Mild	87	8 (9%)	4 (5%)	45 (52%)	30 (34%)
	Mod	53	15 (28%)	5 (9%)	28 (53%)	5 (9%)
	Severe	11	3 (27%)	3 (27%)	5 (45%)	0 (0%)
	16-45	17	2 (12%)	0 (0%)	7 (41%)	8 (47%)
	46-65	134	24 (18%)	12 (9%)	71 (53%)	27 (20%)
			**n=15**	**n=18**	**n=41**	**n=58**
**SE Asia**	Mild	93	11 (12%)	12 (13%)	24 (26%)	46 (49%)
	Mod	34	4 (12%)	6 (18%)	14 (41%)	10 (29%)
	Severe	5	0 (0%)	0 (0%)	3 (60%)	2 (40%)
	16-45	21	2 (10%)	3 (14%)	5 (24%)	11 (52%)
	46-65	111	13 (12%)	15 (14%)	36 (32%)	47 (42%)

Note. AUS=Australia, UK=United Kingdom, SE Asia=South East Asia, Mod=Moderate
