# Supplementary material for: Exploring post acute rehabilitation service use and outcomes for working age stroke survivors (≤65 years) in Australia, UK and South East Asia: data from the international AVERT trial
Source: BMJ Open. 2020 Jun 11;10(6):e035850. doi: 10.1136/bmjopen-2019-035850 (PMC7295421; doi:10.1136/bmjopen-2019-035850)
Supplement: Supplementary data [file bmjopen-2019-035850supp002.pdf]

Supplement 2

S-Table 2. Recommendations for post stroke rehabilitation from Australia, UK and Singapore clinical guidelines during AVERT

Guideline	Recommendation
Australia (NSF, 2005 & 2007)[35,36]	All people admitted to hospital with stroke and who require rehabilitation should be treated in a comprehensive or rehabilitation stroke unit with an interdisciplinary team (Level I)<br><br>If ongoing inpatient rehabilitation is needed, care should be provided in either a stroke rehabilitation unit or a general rehabilitation unit (Grade A; Level I)<br><br>Rehabilitation in the community is equally effective if delivered in the hospital via outpatients, or day hospital, or in the community, and should be offered to all stroke patients as needed (Grade A; Level I)<br><br>Where comprehensive interdisciplinary community rehabilitation services and carer support services are available, early supported discharge services may be provided for people with mild to moderate disability (Level I)
UK (RCP 3rd edition, 2008)[34]	Once a patient is medically investigated, treated and stable, rehabilitation should be delivered by a specialist stroke service (in an inpatient unit, or a day-hospital unit, or at home)<br><br>All patients discharged home directly after acute treatment but with residual problems should be followed up by specialist stroke rehabilitation services<br><br>Domiciliary rehabilitation services should be commissioned as part of an 'early supported discharge' scheme to deliver specialist rehabilitation at home in liaison with inpatient services, as well as in the long-term<br><br>Patients should only be discharged early (before the end of acute rehabilitation) from National Clinical Guideline for Stroke hospital if there is a specialist stroke rehabilitation team able to continue rehabilitation in the community from the day of transfer and if the patient is able to transfer safely from bed to chair, and if other problems can be safely managed at home
Singapore (MoH, 2009)[31]	Stroke patients should receive organized inpatient multidisciplinary rehabilitation (Grade A, Level 1+)<br><br>If able to do so, stroke patients should be encouraged to participate in more intensive rehabilitation particularly in the first six months (Grade A, Level 1+)

Levels of evidence
NSF (2005, 2007)[35,36]
Level I Evidence obtained from a systematic review of all relevant randomised controlled trials. Grade A recommendation: A Body of evidence can be trusted to guide practice

Singapore MoH (2009)[31]
Level 1+: Well conducted meta-analyses, systematic reviews of RCTs, or RCTs with a low risk of bias.
Grade A Recommendation: At least one meta-analysis, systematic review of RCTs, or RCT rated as 1+ + and directly applicable to the target population; or A body of evidence consisting principally of studies rated as 1+, directly applicable to the target population, and demonstrating overall consistency of results
