# Supplementary material for: Exploring post acute rehabilitation service use and outcomes for working age stroke survivors (≤65 years) in Australia, UK and South East Asia: data from the international AVERT trial
Source: BMJ Open. 2020 Jun 11;10(6):e035850. doi: 10.1136/bmjopen-2019-035850 (PMC7295421; doi:10.1136/bmjopen-2019-035850)
Supplement: Author's manuscript [file bmjopen-2019-035850.draft_revisions.pdf]

BMJ Open

BMJ Open is committed to open peer review. As part of this commitment we make the peer review history of every article we publish publicly available.

When an article is published we post the peer reviewers' comments and the authors' responses online. We also post the versions of the paper that were used during peer review. These are the versions that the peer review comments apply to.

The versions of the paper that follow are the versions that were submitted during the peer review process. They are not the versions of record or the final published versions. They should not be cited or distributed as the published version of this manuscript.

BMJ Open is an open access journal and the full, final, typeset and author-corrected version of record of the manuscript is available on our site with no access controls, subscription charges or pay-per-view fees (http://bmjopen.bmj.com).

If you have any questions on BMJ Open's open peer review process please email info.bmjopen@bmj.com

BMJ Open

Post-acute rehabilitation service use and outcomes for working age stroke survivors (≤65 years) in Australia, United Kingdom and South East Asia: Data from the international AVERT trial

Journal:	*BMJ Open*
Manuscript ID	bmjopen-2019-035850
Article Type:	Original research
Date Submitted by the Author:	19-Nov-2019
Complete List of Authors:	Walters, Rosy; Royal Free London NHS Foundation Trust, Physiotherapy ; Florey Institute of Neuroscience and Mental Health - Austin Campus, Stroke<br>Collier, Janice M.; Florey Institute of Neuroscience and Mental Health - Austin Campus, Stroke<br>Braighi Carvalho, Lillian; Florey Institute of Neuroscience and Mental Health - Austin Campus, Stroke<br>Langhorne, Peter; University of Glasgow, Institute of Cardiovascular and Medical Sciences,<br>katijjahbe, Md Ali; Hospital Canselor Tuanku Muhriz, Physiotherapy; Swinburne University of Technology, Health Professions<br>Tan, Dawn; Singapore General Hospital, Department of Physiotherapy<br>Moodie, Marj; Deakin University, Deakin Health Economics<br>Bernhardt, Julie; Florey Institute of Neuroscience and Mental Health - Austin Campus, Stroke
Keywords:	Stroke < NEUROLOGY, REHABILITATION MEDICINE, Organisation of health services < HEALTH SERVICES ADMINISTRATION & MANAGEMENT

SCHOLARONE™
Manuscripts

I, the Submitting Author has the right to grant and does grant on behalf of all authors of the Work (as defined in the below author licence), an exclusive licence and/or a non-exclusive licence for contributions from authors who are: i) UK Crown employees; ii) where BMJ has agreed a CC-BY licence shall apply, and/or iii) in accordance with the terms applicable for US Federal Government officers or employees acting as part of their official duties; on a worldwide, perpetual, irrevocable, royalty-free basis to BMJ Publishing Group Ltd ("BMJ") its licensees and where the relevant Journal is co-owned by BMJ to the co-owners of the Journal, to publish the Work in this journal and any other BMJ products and to exploit all rights, as set out in our licence.

The Submitting Author accepts and understands that any supply made under these terms is made by BMJ to the Submitting Author unless you are acting as an employee on behalf of your employer or a postgraduate student of an affiliated institution which is paying any applicable article publishing charge ("APC") for Open Access articles. Where the Submitting Author wishes to make the Work available on an Open Access basis (and intends to pay the relevant APC), the terms of reuse of such Open Access shall be governed by a Creative Commons licence – details of these licences and which Creative Commons licence will apply to this Work are set out in our licence referred to above.

Other than as permitted in any relevant BMJ Author's Self Archiving Policies, I confirm this Work has not been accepted for publication elsewhere, is not being considered for publication elsewhere and does not duplicate material already published. I confirm all authors consent to publication of this Work and authorise the granting of this licence.

For peer review only

Post-acute rehabilitation service use and outcomes for working age stroke survivors (≤65 years) in Australia, United Kingdom and South East Asia: Data from the international AVERT trial

AUTHORS

Walters, R.[1,2]

Collier, J. M.[1]

Braighi Carvalho, L.[1]

Langhorne, P.[3]

Katijjahbe, M. A.[4,5]

Tan, D.[6]

Moodie, M.[7]

Bernhardt, J.[1]

on behalf of the AVERT Trialists' Collaboration

AFFILIATIONS

[1]Florey Institute of Neuroscience and Mental Health, University of Melbourne, Heidelberg Australia

[2]Physiotherapy, Royal Free London NHS Foundation Trust, London, United Kingdom

[3]Academic Section of Geriatric Medicine, Institute of Cardiovascular and Medical Sciences, University of Glasgow, Glasgow, United Kingdom

[4]Physiotherapy, Hospital Canselor Tuanku Muhriz, Pusat Perubatan UKM, Cheras Kuala Lumpur

[5]Department of Health Professions, Faculty of health, Art and Design, Swinburne University of Technology, Hawthorn, Victoria.

[6]Physiotherapy, Singapore General Hospital, Singapore

[7]Deakin Health Economics, Deakin University, Burwood Australia

*Corresponding author: Julie Bernhardt, 245 Burgundy Street, Heidelberg Australia 3084

julie.bernhardt@florey.edu.au

Ph: 613 9305 7072

For peer review only

Word Count: 3636 words

Tables n=1

Figures n=4

Supplementary Files n=2

Keywords: young stroke, rehabilitation services, return to work, outcome, international

For peer review only

ABSTRACT

Objectives Younger people of working age (≤65 years) need information on age relevant outcomes after stroke. The large international very early rehabilitation trial (AVERT) found outcomes were not different between intervention groups at 12 months. This provided a unique opportunity to describe: (i) baseline demographics; (ii) post-acute rehabilitation pathways; and (iii) 12 month outcomes; disability, community mobility, depression, quality of life and return to work (RTW); from three different regions (Australasia (AUS), South East (SE) Asia, and United Kingdom (UK)).

Design A post hoc descriptive summary exploring the distribution of participants across four post-acute rehabilitation pathways (inpatient rehabilitation (IR), home with community rehabilitation (CR), IR then CR and, home with no rehabilitation); and outcomes at 12 months post stroke.

Setting Hospital stroke units in AUS, UK and SE Asia.

Participants Acute stroke patients (n=2104) recruited within 24 hours.

Interventions Very early intensive mobilisation plus usual care, or usual care alone.

Results Participants ≤65 years, n=668; 99% living independently; and 88% no disability (modified Rankin Score (mRS)=0) prior to stroke. Complete data for 12 month outcomes were n=631 (94%). The proportion receiving IR was higher in AUS than other regions (AUS 52%; UK 25%; SE Asia 23%), whereas the UK had higher CR (UK 65%; AUS 61%; SE Asia 39%). At 12 months, 70% had no or little disability (mRS 0-2), 44% were depressed, 28% rated quality of life (QOL) as poor or worse than death. For those working prior to stroke (n=228), only 57% had RTW. A noteworthy number of working age survivors received no rehabilitation services in the first 3 months post stroke.

Conclusions There was considerable variation in rehabilitation pathways and service use across the three regions. At 12 months there were high rates of depression, poor QOL, and low rates of RTW.

Trial registration Australian New Zealand Clinical Trials Registry ACTRN12606000185561

For peer review only

Article Summary

Strengths and limitations of this study

- Large acute rehabilitation trial provides descriptive data and overview of regional service patterns.
- Little missing data provide high confidence in outcomes.
- Smaller numbers of participant and wide diversity of health services in SE Asia limit insights for this service region.

For peer review only

INTRODUCTION

Evidence suggests stroke incidence in people of working age is on the rise (Bejot et al, 2016). In Australia, New Zealand and the United Kingdom (UK), 25-30% of strokes occur in people of working age (≤65 years).[1-4] With 51,000 strokes/year in Australia[1] and 152,000/year in the UK[5], this equates to an estimated 12,750 and 38,000 strokes in people of working age each year in these countries respectively.

With five-year survival rates the highest for those 50 years and younger[6], stroke survivors of working age may live many years with the physical, psychological and social consequences of stroke. Stroke survivors of working age have unique rehabilitation needs and different expectations of recovery.[7-9] Outcomes pertinent to stroke survivors of working age may include a return to independence, self-reliance, and work.[10, 11] Alongside from the personal impact, the financial burden associated with stroke disability in people of working age, to individuals, carers and the economy is considerable. The cost of lost earnings due to reduced employment from stroke in Australia alone was estimated as $975 million in 2013.[1] Rehabilitation is a time limited, costly, but valued part of post-stroke care.

How stroke services are organised to meet the needs of survivors varies between countries. Service priorities and guideline implementation also vary across different healthcare settings, and according to government health structures, geography and culture.[12] Acute care in organised stroke units is supported by high level evidence, and global efforts to standardise acute medical treatments has improved stroke unit uptake, which typically includes some level of assessment and treatment by a multidisciplinary team. Inter-country or regional differences in rehabilitation access and pathways following acute care are less well understood and therefore a focus of this study.

Post-acute stroke rehabilitation services often include two key models:

- Inpatient Rehabilitation (IR): rehabilitation service delivered in the hospital setting
- Community Rehabilitation (CR): rehabilitation service delivered as an outpatient at a clinic or day hospital or in the patient's own home. This model includes Early Supported Discharge (ESD) which offers early discharge from acute hospital and provision of rehabilitation and support in the home.

For peer review only - http://bmjopen.bmj.com/site/about/guidelines.xhtml

The AVERT trial of very early rehabilitation[13] included 2104 participants from 5 countries (3 regions; Australasia, South East Asia, United Kingdom) who were recruited within 24 hours of stroke, and randomised to two rehabilitation interventions: very early intensive mobilisation plus usual care, or usual care alone. Twelve month disability and health related quality of life outcomes were not different between intervention groups.[14] This large dataset provided a unique opportunity to explore rehabilitation pathways and 12 month outcomes for the subgroup of participants of working age.

Specifically, our aims were:

(i) To describe differences in demographics of the working age stroke population in three regions

(ii) To explore the distribution of these individuals across four post-acute rehabilitation pathways in three regions

(iii) To describe disability, return to work, mood, informal care, mobility and quality of life outcomes for working age people with stroke at 12 months in three regions: disability, community mobility, depression, quality of life and return to work

METHODS

We have defined 'working age stroke' as those aged 18-65 years who are considered to be of working age; as pension age in the UK, Australia & New Zealand is 65-67 years and minimum retirement in Malaysia and Singapore age is 60-62.[15]

Design

This is a post-hoc exploratory descriptive evaluation using data from the AVERT trial. AVERT was a single-blind, randomised controlled trial with a comprehensive economic evaluation in which participants were recruited from 56 acute stroke units between 2006 and 2015. Eligible participants for AVERT were aged 18 years or older, with no significant pre-morbid disability (modified Rankin Score ≤2), with confirmed first or recurrent stroke, admitted to a stroke unit within 24 hours of stroke onset, and medically stable. The AVERT trial method has been described in detail previously[13] with the trial protocol published.[16] Human Research Ethics Committee approval was obtained from all sites and conformed to the Declaration of Helsinki.

For peer review only

Participants or representatives provided individual consent. A stroke consumer provided input throughout the trial planning, training, progress and results as a member of the trial management and executive committees.

We extracted patient demographics, baseline stroke characteristics, discharge location, rehabilitation care, and patient outcomes for participants ≤ 65 years and grouped these into the three regions with similar health and social organisation: Australasia (Australia and New Zealand), United Kingdom (UK) (England, Scotland, Wales, Northern Ireland) and South East (SE) Asia (Singapore and Malaysia).

Demographics and baseline stroke characteristics

Demographics included age, sex, presence of comorbid vascular risk factors, previous stroke, pre-morbid disability (modified Rankin Scale, mRS)[17] walking independence and living arrangements prior to stroke. Baseline stroke characteristics included stroke severity (National Institute of Health Score (NIHSS) score: mild (NIHSS 1–7), moderate (8–16), and severe (>16)[18] and stroke type (Oxfordshire Community Stroke Type Project classification).[19] Baseline walking used the gait component of the Mobility Scale for Acute Stroke[20] which measures the amount of assistance and independence with walking 10m indoors with or without gait aid using a 6-point scale ranging from 1 (unable) to 6 (independent).

Post-acute discharge pathways

Discharge to home and rehabilitation (inpatient and community) data were collected at both 3 and 12 months by face-to-face interview (blinded assessor) with participants and/or carers, with supporting documentation sourced from health care providers. Discharge to supported care (nursing home, supported accommodation) was not included as small numbers were expected. Our focus was on determining resources used, i.e., location of care (hospital, community, home) and days of rehabilitation, rather than on the content of the therapy itself. Rehabilitation data collected at 3 months were used to best represent service delivery relating to the initial stroke event. Four post-acute discharge pathways, which include details of rehabilitation service use, were defined a priori (Figure 1).

<Insert Figure 1 about here>

For peer review only

12 month outcomes

12 month outcomes are reported for the four post-acute discharge pathways. We selected 12 month outcomes which included measures from each domain of The International Classification of Functioning Disability and Health Framework.[21]

Disability: The modified Rankin Scale (mRS)[17] is an ordinal scale ranging from 0 (no disability) to 5 (severe disability) or 6 (death). The proportion with favourable outcome defined as mRS scores 0-2 (no or minimal disability), is reported here.

Community mobility: We used the Rivermead Motor Assessment Gross Function tool (a 13-point scale[22]) reliable in stroke[23] to measure community mobility at 3 and 12 months. We report the proportion achieving a score of 10-13 at 12 months, which indicates an ability to walk independently outside the home.[24]

Work participation: Of those working prior to stroke, we report the proportion of stroke survivors who returned to work at 12 months in any capacity (part time or full time).

Quality of life: The Assessment of Quality of Life Measure (AQoL-4D) comprises 15 items over 5 subscales: independent living, social relationships, physical senses, psychological well-being, and illness.[25] The AQoL utility score, which refers to the value people place on their Health Related Quality of Life (HRQoL), ranges from −0.04 (state worse than death), death (0.0) through to 1.0 (excellent).[26] We report the proportion of stroke survivors scoring *poor* or *worse than death* (score -0.04-0.4) at 12 months.

Depression: We extracted depression data from the Irritability Depression and Anxiety (IDA) scale and report the proportion with 'Borderline' (score 4-6) or 'Morbid' depression (score >7)[27] at 12 months.

Informal care: We report the proportion of stroke survivors requiring informal care at 12 months; defined as the participant requiring help with activities of daily living beyond that provided by formal support services.

For peer review only

Analyses

Baseline, rehabilitation service use and 12 month outcome data were presented using descriptive analysis (n, %) using STATA IC for all analyses. Median and interquartile ranges are presented for age, baseline stroke severity (NIHSS score) and length of stay. We describe pathway use by baseline stroke severity (mild, moderate and severe), and by younger (18-45 years) and older (46-65) age.

RESULTS

In the AVERT trial, 668 participants were ≤65 at time of stroke; 363 participants were from Australasia, 161 from the UK and 144 from SE Asia. Characteristics of these participants are presented in Table 1. The median age of participants was similar across the three regions (58 years) with a high proportion of men (73%). Prior to stroke, 99% of participants were living independently, 88% had no disability (mRS=0), 97% were walking without a gait aid and 62% were working. The highest proportion of participants with hypertension, diabetes mellitus and ischaemic heart disease were from SE Asia. Nearly two thirds of the people in SE Asia had lacunar strokes. The UK had the highest proportion of current smokers.

Table 1: Premorbid & baseline stroke characteristics of participants ≤65 by region

	Australasia n = 363	UK n=161	SE Asia n = 144	Total n = 668
**Baseline Characteristics**				
**Age**, y, median (IQR)	57.6 (49.5-62.3)	58.4 (51.5-62.6)	57.6 (51.8-61.9)	57.9 (50.3-62.2)
range	19.93-65.99	37.53-65.98	16.4-65.54	16.4-65.99
**Sex**				
Female	98 (27.0%)	56 (34.8%)	50 (34.7%)	204 (30.5%)
Male	265 (73.0%)	105 (65.2%)	94 (65.3%)	464 (69.5%)
**Risk factors**				
Hypertension	198 (54.5%)	84 (52.2%)	105 (72.9%)	387 (57.9%)
Hypercholesterolemia	119 (32.8%)	67 (41.6%)	54 (37.5%)	240 (35.9%)
Diabetes mellitus	68 (18.7%)	31 (19.3%)	61 (42.4%)	160 (24.0%)
Ischemic heart disease	58 (16.0%)	20 (12.4%)	24 (16.7%)	102 (15.3%)
Atrial fibrillation	36 (9.9%)	11 (6.8%)	11 (7.6%)	58 (8.7%)
**Smoking**				
Never Smoked	139 (38.3%)	51 (31.7%)	78 (54.2%)	268 (40.1%)

For peer review only

Smoker*	129 (35.5%)	70 (43.5%)	49 (34.0%)	248 (37.1%)
Ex-Smoker*	91 (25.1%)	39 (24.2%)	16 (11.1%)	146 (21.9%)
Unknown	4 (1.1%)	1 (<1%)	1 (<1%)	6 (<1%)
**Premorbid data**				
**modified Rankin Scale**				
0	319 (87.9%)	133 (82.6%)	135 (93.8%)	587 (87.9%)
1	26 (7.2%)	17 (10.6%)	5 (3.5%)	48 (7.2%)
2	18 (5.0%)	11 (6.8%)	4 (2.8%)	33 (4.9%)
**Independent walking without aid**	356 (98.1%)	151 (93.8%)	139 (96.5%)	646 (96.7%)
**Living arrangement at time of admission**				
Home alone	52 (14.3%)	38 (23.6%)	22 (15.3%)	112 (16.8%)
Home with someone	310 (85.4%)	122 (75.8%)	121 (84.0%)	553 (82.8%)
Supported accommodation	1 (<1%)	0 (0%)	1 (<1%)	2 (<1%)
Other	0 (0%)	1 (<1%)	0 (0%)	1 (<1%)
**Employment Status**				
Working at time of stroke	238 (65.6%)	89 (55.3%)	86 (59.7%)	413 (61.8%)
Full Time	188 (79.0%)	66 (74.2%)	73 (84.9%)	327 (79.2%)
Part Time	50 (21.0%)	23 (25.8%)	13 (15.1%)	86 (20.8%)
**Baseline stroke**				
**First stroke, n (%)**	315 (86.8%)	141 (87.6%)	112 (77.8%)	568 (85.0%)
NIHSS score, median (IQR)	6 (4-11)	6 (4-11)	5 (4-8)	6 (4-10)
Mild (1-7)	213 (58.7%)	93 (57.8%)	99 (68.8%)	405 (60.6%)
Moderate (8-16)	107 (29.5%)	56 (34.8%)	39 (27.1%)	202 (30.2%)
Severe (>16)	43 (11.9%)	12 (7.5%)	6 (4.2%)	61 (9.1%)
**Stroke type**				
Oxfordshire Stroke Classification				
Total anterior circulation infarct	61 (16.8%)	28 (17.4%)	8 (5.6%)	97 (14.5%)
Partial anterior circulation infarct	109 (30.0%)	55 (34.2%)	21 (14.6%)	185 (27.7%)
Posterior circulation infarct	53 (14.6%)	11 (6.8%)	7 (4.9%)	71 (10.6%)
Lacunar infarct	80 (22.0%)	50 (31.1%)	91 (63.2%)	221 (33.1%)
Haemorrhage				
Intracerebral haemorrhage	60 (16.5%)	17 (10.6%)	17 (11.8%)	94 (14.1%)
**Baseline walking** (Mobility Scale for Acute Stroke walking score)				
Independent	45 (12.4%)	31 (19.3%)	12 (8.3%)	88 (13.2%)
Supervised or assisted	200 (55.1%)	61 (37.9%)	83 (57.6%)	344 (51.5%)
Unable to walk	117 (32.2%)	69 (42.9%)	49 (34.0%)	235 (35.2%)
Missing or unknown	1 (<1%)	0 (0%)	0 (0%)	1 (<1%)

Data are n (%) or median (IQR) NIHSS=National Institute of Health Stroke Scale
*Smoker defined as a current smoker or participant who had quit smoking in the past 2 years, and an ex-smoker as a participant who had quit smoking more than 2 years ago

For peer review only

Rehabilitation Pathways

The pathways of rehabilitation service use to 3 months, and 12 month outcome summaries for each region are shown in Figures 2-4. Of the 668 participants, 94% (n=631/668) followed one of the four common pathways of post-acute discharge. Most were discharged home (n=384/668, 57%) or to inpatient rehabilitation (n=261/668, 39%). Fourteen participants died in acute care. The discharge destination of the remaining participants was a nursing home (n=5), with missing discharge (n=4) and community rehabilitation (n=14) data.

<Insert Figure 2 about here>
<Insert Figure 3 about here>
<Insert Figure 4 about here>

In the first 3 months post stroke, 67% of participants received rehabilitation (IR or CR) services. Only 8% were still receiving rehabilitation services at 12 month follow up. The proportion receiving IR was higher in Australasia than other regions (Australasia 52%; UK 25%; SE Asia 23%). The length of stay in IR was longest in UK (median 35 days) and shortest in SE Asia (median 18 days) and the UK had higher participation in CR than other regions (UK 65%; Australasia 61%; SE Asia 39%). It was most common for UK stroke survivors to go home from acute care and receive CR (48%). For those discharged home from acute care, the length of stay was similar in the three regions (median 4-5 days). The number of working age survivors who went home after a short acute care stay and received no rehabilitation (IR or CR) was unexpectedly high (Australasia 25%; UK 22%; SE Asia 40%).

The proportion of stroke survivors with mild, moderate and severe stroke, grouped by younger versus older age, and their service use pathways by regions is shown in Supplement 1. As could be anticipated, the highest proportion of working age survivors who did not receive rehabilitation were those with mild stroke (40%, n=156/389) compared to those with moderate (12%, n=24/200) or severe (4%, n=2/52) stroke. Less expected was our finding that 38% (n=35/92) of the younger

For peer review only

age group aged 18-45 years did not receive any rehabilitation on discharge from acute care.

12 month outcomes

Of the 631 working age stroke survivors included in the four discharge pathways, 2% (n=14/631) had died by 12 month follow up. Some 70% (n=444/631) had a favourable outcome (mRS 0-2) reporting little or no disability (mRS missing data 3%, n=20/631), yet 34% (n=217/631) were still receiving informal care. 79% (n=497/631) were community ambulators (Rivermead Mobility>9) but only 57% of those working prior to stroke (n=228/398) had returned to work. The proportion who had returned to work at 12 months was similar across the three regions: Australasia 61%, SE Asia 57%, UK 48%. 28% (n=174/631) had a HRQoL rated as poor or worse than death: UK 34%, SE Asia 27%, and Australasia 25%. Borderline or morbid depression was reported by 44% (n=276/631), with the highest proportion in the UK 52% and the lowest proportion in SE Asia 31%. The largest proportion of missing data was for depression as some stroke survivors were unable to complete the questionnaire (IDA, 10%, n=65/631).

DISCUSSION

This report of rehabilitation service use and 12 month outcomes of working age stroke survivors across three geographical regions with differing health services using this well characterised and large dataset has yielded some unique findings. Interestingly, while countries in all three regions in this study have universal healthcare as a core principle of their healthcare system, we saw considerable variation in rehabilitation pathways and service use across the regions. For example, around 50% of working age stroke survivors in Australasia received inpatient rehabilitation, compared with around 25% of stroke survivors in the UK or SE Asia. In the case of SE Asia, many went straight home from acute stroke care and 40% of these individuals had no further rehabilitation. Whether this reflects differences in access to inpatient rehabilitation services between these regions, or other factors, was not examined in this study. The SE Asia data from AVERT included data from Singapore and Malaysia. Singapore is classified as an ‘advanced economy’ and Malaysia as a ‘developing economy’ (International Monetary Fund, 2015). The Malaysian Clinical Practice Guidelines 2012[28] do not address care beyond the acute

For peer review only - http://bmjopen.bmj.com/site/about/guidelines.xhtml

stroke unit. Indeed, all working age stroke survivors in SE Asia admitted to inpatient rehabilitation (n=33) were from Singapore. It should be noted that Singapore stroke guidelines[29] recommend that 'stroke patients should receive organised inpatient multidisciplinary rehabilitation' (Supplement 2). In this dataset, only 51% (n=33/65) of participants from Singapore received inpatient rehabilitation.

The larger proportion of working age stroke survivors from SE Asia who went home with no rehabilitation compared to those in Australasia or UK may be partly explained by a difference in stroke severity. However, median baseline stroke severity for this group was similar across the three regions (median NIHSS 5 SE Asia; 3 AUS; 4 UK). It may reflect the access to community rehabilitation services in the region. There is currently no protocol in Malaysia which specifically addresses the transfer of care for long-term post stroke management at community level.[30] A recent study of post stroke management in Malaysia[30] found that almost one third of patients (31%) were not referred to any rehabilitation facility. They cited the lack of awareness among physicians regarding the role of neuro rehabilitation and the lack of co-ordination of post stroke care beyond the acute phase as contributing factors. It is possible that participants were offered a referral for outpatient rehabilitation services but declined due to the cost incurred, or that the group included overseas participants who were not eligible for local rehabilitation services.

In contrast with SE Asia, the lower rates of inpatient rehabilitation in the UK appear to be offset by the higher use of community rehabilitation, with 48% of UK working age stroke survivors discharged home from acute care with community rehabilitation. Early supported discharge (ESD) services have been a focus of service development in the UK over a number of years. The ESD model has been shown to reduce length of stay and long-term dependency at 12 months in people with mild to moderate stroke severity.[31] We found that the majority of stroke survivors who received community rehabilitation were those with mild and moderate stroke, consistent with entry criteria for an ESD service. Only 20% of survivors in Australasia went directly home with community rehabilitation. Although ESD has been recommended in the Australian Clinical Practice Guidelines[32] since 2005 (Supplement 2), if we assume

For peer review only

that episodes of community rehabilitation reflect ESD, so far this model does not appear to be common practice in Australia. A challenge with interpreting community rehabilitation data is that it is often poorly described, and the recommended comprehensive ESD may not be implemented.

We were surprised to find that nearly a quarter of stroke survivors in the UK and Australasia had no rehabilitation services in the first three months post stroke. None of these individuals had severe stroke, so perceived absence of 'rehabilitation potential' would not explain this finding. In the UK and Australasia, a large proportion (30-47%) of the younger age group 18-45, went home with no rehabilitation. The same pattern was present for those with mild stroke at baseline, with 34-38% of these individuals in UK and Australasia not receiving rehabilitation. This finding may relate to professionals' expectations that younger survivors and those with mild stroke will spontaneously recover without services. The need for rehabilitation services may not have been identified; 'mild' symptoms may not be apparent in the acute setting, and some, such as mood disturbance, may have a delayed onset. Stroke is a sudden, life-changing event for a young adult, the effects of which may not be anticipated during acute care. A systematic review of qualitative research reports a young adult stroke describing; 'When you're in hospital, you don't really think about how your life is going to be when you leave. You think that you'll just carry on as before, but you don't. You get home and that's when the trauma starts'.[33] A flexible model of stroke service use and follow-up is indicated, such as the 'point of contact with specialist services' recommended in Parkinson's Disease,[2] to provide ongoing support for working age stroke.

Across all three regions, we found that 12 month outcomes for those who did not receive rehabilitation were less than optimal. Whether an episode of rehabilitation would have improved these outcomes is unknown. What is perhaps most pertinent is whether the rehabilitation needs of patients in the acute phase of care were formally assessed. This was not a focus of the AVERT trial. Advances in post stroke interventions, such as thrombolysis and endovascular clot retrieval, have led to ongoing reductions in acute length of stay post-stroke.[34, 35] Less time in hospital may limit time for multidisciplinary assessment and identification of post-discharge needs.

For peer review only

With a median acute length of stay for participants discharged directly home of 4-5 days in each of the three regions, it may be difficult for rehabilitation needs to be adequately assessed. The need for standardised assessment of patients in acute care for stroke rehabilitation has led to the development of Assessment for Rehabilitation: Pathway and Decision-Making Tool.[36] It is now recommended that all stroke survivors who are not for palliative care should be considered for rehabilitation.[36] Our data suggests that for a significant number of working age stroke survivors, the acute care stay may be their only contact with specialised medical and allied health stroke services, and that those under 45 years and those with mild stroke may be vulnerable to a lack of coordinated specialist post discharge care.

In this study, 70% reported little to no disability at 12 months (favourable outcome mRS 0-2), which is consistent with previous research of those with working age stroke.[37-39] A large number of working age stroke survivors reported low mood (44% with borderline or morbid depression) and poor quality of life (28% rated HRQoL as poor or worse than death; missing data n=30). Only 7% of healthy people the same age report this level of HRQoL,[26] suggesting the need for research to determine if targeted interventions can improve QoL in working age stroke survivors. Depression has been shown to be independently associated with low HRQoL in working age stroke survivors[40] and may be improved with psychosocial, neuropsychological or pharmacological interventions.[4] The Australian Stroke Guidelines[41] recommend routine assessment of mood disturbance post stroke, and the UK[4] has identified that service improvements in the organisation and delivery of psychological services for stroke survivors are needed. The psychosocial needs of working age stroke survivors are commonly reported as being 'unmet'[42-44] and surveillance and rehabilitation programs that specifically address these issues appear needed.

The proportion of working age stroke survivors (57%) in this study who had returned to work at 12 months was low but consistent with previous research.[45, 46] Poor rates of return to work despite good functional outcomes have been reported. For example, Varona and colleagues[39] evaluated long-term outcomes in younger adults after first ischemic stroke and found while 90% were independent, only 53% had returned to work. Return to work post stroke is complex and multifactorial[47, 48], with optimal interventions to improve return to work outcomes for people of working age

For peer review only

being poorly understood.[49] A recent systematic review[46] concluded that either specialised vocational rehabilitation, conventional stroke rehabilitation or their combination is needed to increase return-to-work rates and improve the quality of life for stroke survivors of working age.

Study limitations

We elected to explore data for all stroke survivors of 'working age' (i.e ≤65 years) which is consistent with other working age definitions. However, in our study population, only 62% of participants were in formal paid employment prior to their stroke. We acknowledge that some people continue to work after age 65, and that the absence of information about unpaid, productive work is a limitation of the data. Further, the absence of information about the specific goals or types of rehabilitation has limited our ability to describe in more detail the rehabilitation services on offer. Nevertheless, the data available from three separate geographical regions offer unique insights into who gets into rehabilitation, and what type, but not why. We elected not to run exploratory analyses on these data given the very small numbers of patients in sub-groups of interest, such as those of younger age (<45 years), different genders, or with mild stroke. Finally, younger stroke survivors live many years with post stroke disability and their needs may change over time. This study reflects only the first 12 months after stroke.

Conclusions

Despite the majority of working age stroke survivors in this study receiving rehabilitation services within the first three months, many reported low mood, poor QoL and failure to return to work at 12 months. Over a quarter (28%) of working age stroke survivors received no rehabilitation services. Younger individuals and those with mild stroke may not be offered rehabilitation yet report sub-optimal outcomes in the longer term. At present, there is little research targeting the needs of younger survivors or indicating benefit, this is an avenue for further enquiry. Further research into the most effective and cost-effective rehabilitation service use pathway for young adults with varying severity of stroke is needed to assist in the development of specific evidence-based guidelines to inform service delivery.

Acknowledgements

For peer review only - http://bmjopen.bmj.com/site/about/guidelines.xhtml

We thank the AVERT Collaboration investigators for their hard work and dedication, and the participants in this trial and their supporters. The Florey Institute of Neuroscience and Mental Health acknowledges the support received from the Victorian Government via the Operational Infrastructure Support Scheme.

Collaborators

The AVERT Trialists' Collaboration[13]

Author Contributions

RS and JB conceived the study and all authors contributed to the study design. Rehabilitation services data was devised by MM, data was managed by JC and collected by the AVERT Trialists' Collaboration. RS performed data analysis. Interpretation was provided by JB, JMC, LBC, PL, KMA and DT. RS was the primary writer of the manuscript, with revisions and intellectual content from all authors. All authors provided final approval of the version to be published.

Funding

The AVERT trial was initially supported by the National Health and Medical Research Council (NHMRC) of Australia (grant numbers 386201 and 1041401). Additional funding was received from Chest Heart and Stroke Scotland (Res08/A114), Northern Ireland Chest Heart and Stroke, Singapore Health (SHF/FG401P/2008), the UK Stroke Association (TSA2009/09), and the UK National Institute of Health Research (HTA Project 12/01/16). NHMRC fellowship funding was provided to JB (1058635, 1154904). JB also received fellowship funding from the Australia Research Council (0991086) and the National Heart Foundation. RW received a Clinical Stipend from the NHMRC Centre of Research Excellence in Stroke Rehabilitation and Brain Recovery (1077898).

Competing interests

Ms Walters reports a clinical stipend from a NHMRC grant. Dr. Langhorne reports grants from NIHR, Stroke Association, Chest Heart and Stroke Scotland. Dr Bernhardt reports grants from the NHMRC, Stroke Association, Chest Heart and Stroke Scotland, Northern Ireland Chest Heart and Stroke, NIHR, ARC and NHF. Dr Moodie reports grants from NHMRC. Drs Tan and Bernhardt reports grants from

For peer review only

Singapore Health. Drs Collier, Braighi Carvalho and Katijjahbe report nothing to disclose.

Patient consent for publication

Consent was obtained for de-identified publication of group data.

Data sharing statement

Data may be shared by writing to the corresponding author to obtain details of procedures and processes.

Open access

This is an open access article distributed in accordance with the Creative Commons Attribution Non Commercial (CC BY-NC 4.0) license, which permits others to distribute, remix, adapt, build upon this work non-commercially, and license their derivative works on different terms, provided the original work is properly cited, appropriate credit is given, any changes made indicated, and the use is non-commercial. See: http:// creativecommons. org/ licenses/ by- nc/ 4. 0/.

For peer review only

References

1. Deloitte Access Economics. The economic impact of stroke in Australia. 2013
2. National Institute for Health and Care Excellence. NICE Guidance. Parkinson's Disease. 2018
3. Tobias M, Cheung J, Carter K, Anderson C, Feigin VL. Stroke surveillance: population based estimates and projections for New Zealand. *Aust. N. Z. J. Public Health*. 2007;31:520-525. doi: 10.1111/j.1753-6405.2007.00136.x
4. Royal College of Physicians. National clinical guideline for stroke 2016. Prepared by the Intercollegiate Stroke Working Party. 2016;Fifth Edition
5. Townsend N, Wickramasinghe K, Bhatnagar P, Smolina K, Nichols M, Leal J, Luengo-Fernandez R, Rayner M. Coronary heart disease statistics 2012. 2012
6. Smajlovic D, Kojic B, Sinanovic O. Five-year survival-after first-ever stroke. *Bosn. J. Basic Med. Sci.* 2006;6:17-22. doi: 10.17305/bjbms.2006.3138
7. Morris R. The psychology of stroke in young adults: The roles of service provision and return to work. *Stroke Res Treatment*. 2011:534812. doi: 10.4061/2011/534812
8. Dale Stone S. Reactions to invisible disability: the experiences of young women survivors of hemorrhagic stroke. *Disabil. Rehabil.* 2005;27:293-304. doi: 10.1080/09638280400008990
9. Teasell RW, McRae MP, Finestone HM. Social issues in the rehabilitation of younger stroke patients. *Arch. Phys. Med. Rehabil.* 2000;81:205-209. doi: 10.1016/s0003-9993(00)90142-4

10. Dixon G, Thornton EW, Young CA. Perceptions of self-efficacy and rehabilitation among neurologically disabled adults. *Clinical Rehabilitation.* 2007;21:230-240. doi: 10.1177/0269215506071784
11. Lawrence M, Kinn S. Needs, priorities, and desired rehabilitation outcomes of family members of young adults who have had a stroke: findings from a phenomenological study. *Disabil. Rehabil.* 2013;35:586-595. doi: 10.3109/09638288.2012.711895
12. Putman K, De Wit L. European comparison of stroke rehabilitation. *Top. Stroke Rehabil.* 2009;16:20-26. doi: 10.1310/tsr1601-20
13. The AVERT Trial Collaboration group, Bernhardt J, Langhorne P, Lindley RI, Thrift AG, Ellery F, Collier J, Churilov L, Moodie M, Dewey H, Donnan G. Efficacy and safety of very early mobilisation within 24 h of stroke onset (AVERT): a randomised controlled trial. *The Lancet*. 2015;386:46-55. doi: 10.1016/S0140-6736(15)60690-0
14. Cumming TB, Churilov L, Collier J, Donnan G, Ellery F, Dewey H, Langhorne P, Lindley RI, Moodie M, Thrift AG, Bernhardt J, on behalf of the AVERT Trial Collaboration Group. Early mobilization and quality of life after stroke. Findings from AVERT. *Neurology*. 2019;93:e1-e12. doi: 10.1212/WNL.0000000000007937
15. The Statutes of the Republic of Singapore. Retirement and Re-Employment Act (chapter 274A). Revised Edition 2012:1-30
16. Langhorne P, Wu O, Rodgers H, Ashburn A, Bernhardt J. A Very Early Rehabilitation Trial after stroke (AVERT): a phase III, multicentre, randomised controlled trial. *Health Technol. Assess.* 2017;21:1-120. doi: 10.3310/hta21540
17. Van Swieten J, Koudstaal P, Visser M, Schouten H, Van Gijn J. Interobserver agreement for the assessment of handicap in stroke patients. *Stroke*. 1988;19:604-607. doi: 10.1161/01.str.19.5.604
18. Briggs DE, Felberg RA, Malkoff MD, Bratina P, Grotta JC. Should mild or moderate stroke patients be admitted to an intensive care unit? *Stroke*. 2001;32:871-876. doi: 10.1161/01.str.32.4.871
19. Bamford J, Sandercock P, Dennis M, Warlow C, Burn J. Classification and natural history of clinically identifiable subtypes of cerebral infarction. *The Lancet*. 1991;337:1521-1526. doi: 10.1016/0140-6736(91)93206-o
20. Simondson JA, Goldie P, Greenwood KM. The Mobility Scale for Acute Stroke Patients: concurrent validity. *Clin. Rehabil.* 2003;17:558-564. doi: 10.1191/0269215503cr650oa
21. World Health Organization. *International Classification of Functioning, Disability and Health: ICF*. World Health Organization; 2001.
22. Lincoln N, Leadbitter D. Assessment of motor function in stroke patients. *Physiotherapy*. 1979;65:48-52.
23. Adams S, Pickering R, Ashburn A, Lincoln N. The scalability of the Rivermead Motor Assessment in non-acute stroke patients. *Clin. Rehabil.* 1997;11:52-59. doi: 10.1177/026921559701100108
24. Cumming TB, Thrift AG, Collier JM, Churilov L, Dewey HM, Donnan GA, Bernhardt J. Very early mobilization after stroke fast-tracks return to walking: further results from the phase II AVERT randomized controlled trial. *Stroke*. 2011;42:153-158.

For peer review only

25. Hawthorne G, Richardson J, Osborne R. The Assessment of Quality of Life (AQoL) instrument: a psychometric measure of health-related quality of life. *Qual. Life Res.* 1999;8:209-224. doi: 10.1023/a:1008815005736
26. Hawthorne G, Osborne R. Population norms and meaningful differences for the Assessment of Quality of Life (AQoL) measure. *Aust. N. Z. J. Public Health*. 2005;29:136-142. doi: 10.1111/j.1467-842x.2005.tb00063.x
27. Snaith RP, Constantopoulos AA, Jardine MY, McGuffin P. A clinical scale for the self-assessment of irritability. *Br. J. Psychiatry*. 1978;132:164-171. doi: 10.1192/bjp.132.2.164
28. Malaysian Society of Neurosciences, Academy of Medicine Malyasia, Ministry of Health Malaysia. Clinical Practice Guidelines. Management of Ischaemic Stroke. 2nd Edition 2012:1-64
29. Ministry of Health. Stroke and Transient Ischaemic Attacks Assessment, Investigation, Immediate Management and Secondary Prevention. *MOH Clinical Practice Guidelines 2/2009*. 2009
30. Abdul Aziz AF, Ali MF, Yusof MF, Che' Man Z, Sulong S, Aljunid SM. Profile and outcome of post stroke patients managed at selected public primary care health centres in Peninsular Malaysia: A retrospective observational study. *Sci. Rep.* 2018;8:17965. doi: 10.1038/s41598-018-36154-0
31. Langhorne P, Baylan S, Early Supported Discharge Trialists. Early supported discharge services for people with acute stroke. *Cochrane Database Syst. Rev.* 2017;7:CD000443. doi: 10.1002/14651858.CD000443.pub4
32. National Stroke Foundation. Clinical Guidelines for Stroke Rehabilitation and Recovery. 2005
33. Lawrence M. Young adults' experience of stroke: a qualitative review of the literature. *Br. J. Nurs.* 2010;19:241-248. doi: 10.12968/bjon.2010.19.4.46787
34. Australian Institute of Health and Welfare (AIHW). Heart, stroke and vascular diseases—Australian facts 2004. *Cardiovascular Disease Series No 22*. 2004
35. Australian Institute of Health and Welfare (AIHW). Stroke and its management in Australia: an update. *Cardiovascular Disease Series*. 2013:158
36. Australian Stroke Coalition Rehabilitation Working Group. Assessment for rehabilitation: pathway and decision-making tool 2012. 2012;Melbourne: Australian Stroke Coalition
37. Knoflach M, Matosevic B, Rucker M, Furtner M, Mair A, Wille G, Zangerle A, Werner P, Ferrari J, Schmidauer C, Seyfang L, Kiechl S, Willeit J, Austrian Stroke Unit Registry Collaborators. Functional recovery after ischemic stroke-a matter of age: data from the Austrian Stroke Unit Registry. *Neurology*. 2012;78:279-285. doi: 10.1212/WNL.0b013e31824367ab
38. Leys D, Bandu L, Henon H, Lucas C, Mounier-Vehier F, Rondepierre P, Godefroy O. Clinical outcome in 287 consecutive young adults (15 to 45 years) with ischemic stroke. *Neurology*. 2002;59:26-33. doi: 10.1212/wnl.59.1.26
39. Varona JF, Bermejo F, Guerra JM, Molina JA. Long-term prognosis of ischemic stroke in young adults. Study of 272 cases. *J. Neurol.* 2004;251:1507-1514. doi: 10.1007/s00415-004-0583-0
40. Naess H, Waje-Andreassen U, Thomassen L, Nyland H, Myhr KM. Health-related quality of life among young adults with ischemic stroke on long-term follow-up. *Stroke*. 2006;37:1232-1236. doi: 10.1161/01.Str.0000217652.42273.02

For peer review only - http://bmjopen.bmj.com/site/about/guidelines.xhtml

41. Stroke Foundation. Clinical Guidelines for Stroke Management. Living Guidelines. https://informme.org.au/en/Guidelines/Clinical-Guidelines-for-Stroke-Management. 2019. Accessed 12 November 2019:Chapter 6.
42. Kersten P, Low JT, Ashburn A, George SL, McLellan DL. The unmet needs of young people who have had a stroke: results of a national UK survey. *Disabil. Rehabil.* 2002;24:860-866. doi: 10.1080/09638280210142167
43. Stroke Foundation. National Stroke Audit. Rehabilitation Services Report 2018. 2018
44. Shipley J, Luker J, Thijs V, Bernhardt J. How can stroke care be improved for younger service users? A qualitative study on the unmet needs of younger adults in inpatient and outpatient stroke care in Australia. *Disabil. Rehabil.* 2019;Jan 7:1-8. doi: https://doi.org/10.1080/09638288.2018.1534278
45. Daniel K, Wolfe CD, Busch MA, McKevitt C. What are the social consequences of stroke for working-aged adults?: A systematic review. *Stroke*. 2009;40:e431-e440. doi: 10.1161/STROKEAHA.108.534487
46. Wei X-J, Liu X-F, Fong KN. Outcomes of return-to-work after stroke rehabilitation: A systematic review. *Br. J. Occup. Ther.* 2016;79:299-308. doi: 10.1177/0308022615624710
47. Glozier N, Hackett ML, Parag V, Anderson CS. The influence of psychiatric morbidity on return to paid work after stroke in younger adults: the Auckland Regional Community Stroke (ARCOS) Study, 2002 to 2003. *Stroke*. 2008;39:1526-1532. doi: 10.1161/strokeaha.107.503219
48. Hackett ML, Glozier N, Jan S, Lindley R. Returning to paid employment after stroke: the Psychosocial Outcomes In StrokE (POISE) cohort study. *PLoS One*. 2012;7:e41795. doi: 10.1371/journal.pone.0041795
49. Baldwin C, Brusco NK. The effect of vocational rehabilitation on return-to-work rates post stroke: a systematic review. *Top. Stroke Rehabil.* 2011;18:562-572. doi: 10.1310/tsr1805-562
50 National Stroke Foundation. *Clinical guidelines for acute stroke management*. Melbourne 2007.
51. Intercollegiate Stroke Working Party. *National Clinical Guidelines for Stroke (Third Edition)*. London: Royal College of Physicians; 2008.

For peer review only

Figure Legends

Figure 1 Four post-acute discharge pathways: Inpatient rehabilitation only; inpatient rehabilitation followed by community rehabilitation; home with community rehabilitation; and home with no community rehabilitation

Note. Pathway does not include discharge to supported accommodation.

Figure 2 Rehabilitation service use and outcomes of participants ≤ 65 years in Australasia

Participants in IR at 3 months n=28 (7.7%); at 12months n=3 (<1%)
Participants receiving CR at 3 months n=1 (<1%), at 12 months n=39 (10.7%)
Participants who had not been discharged from their initial acute admission or were still in IR at the time of 3-month follow-up were allocated to the appropriate pathway using CR data collected at 12 months.
LOS (Length of Stay), Age, NIHSS data are median (IQR)
Depression (IDA scale), HRQoL (AQoL-4D), Community ambulator defined as Rivermead Motor Assessment Gross Function >9
12 month outcomes data are % of group, except Return to Work data which are % of those working prior to stroke
Missing 12 month outcomes data: Unknown Missing n=10/69 (14.5%); Alive Missing n=59/69 (85.5%)

Figure 3 Rehabilitation service use and outcomes of participants ≤ 65 years in United Kingdom

Participants in inpatient rehabilitation at 3 months n=8 (5.0%); at 12 months n=1 (<1%)
Participants receiving community rehabilitation services at 3months n=1 (<1%), at 12 months n=11 (6.8%)
Participants who had not been discharged from their initial acute admission or were still in IR at the time of 3 month follow-up were allocated to the appropriate pathway using CR data collected at 12 months.
LOS (Length of Stay), Age, NIHSS data are median (IQR)
Depression (IDA scale), HRQoL (AQoL-4D), Community ambulator defined as Rivermead Motor Assessment Gross Function >9
12 month data are % of group, except Return to Work data which are % of participants in group working prior to stroke
Missing 12 month outcomes data: Unknown Missing n=15/71 (21.1%); Alive Missing n=56/71 (78.9%)

Figure 4 Rehabilitation service use and outcomes of participants ≤ 65 years in South East Asia

Participants in inpatient rehabilitation at 3 months n=0; at 12 months n=0
Participants receiving community rehabilitation services at 3 months n=2 (1.4%), at 12 months n=0
Participants who had not been discharged from their initial acute admission or were still in IR at the time of 3 month follow-up were allocated to the appropriate pathway using CR data collected at 12 months.
LOS (Length of Stay), Age, NIHSS data are median (IQR)
Depression (IDA scale), HRQoL (AQoL-4D), Community ambulator defined as Rivermead Gross Motor Function >9
12 month data are % of group, except Return to Work data which are % of participants in group working prior to stroke
Missing 12 month outcomes data: Unknown Missing n=6/40 (15%); Alive Missing n=34/40 (85%)

For peer review only

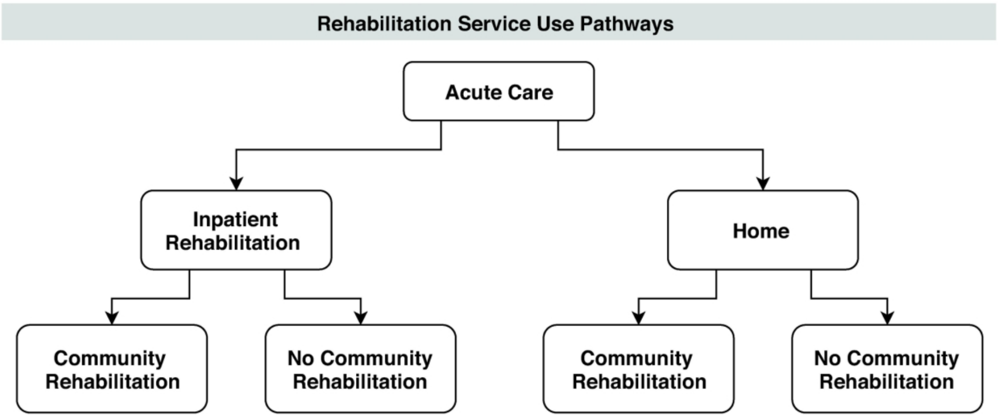

Figure 1 Four post-acute discharge pathways: Inpatient rehabilitation only; inpatient rehabilitation followed by community rehabilitation; home with community rehabilitation; and home with no community rehabilitation
Note. Pathway does not include discharge to supported accommodation.

173x80mm (300 x 300 DPI)

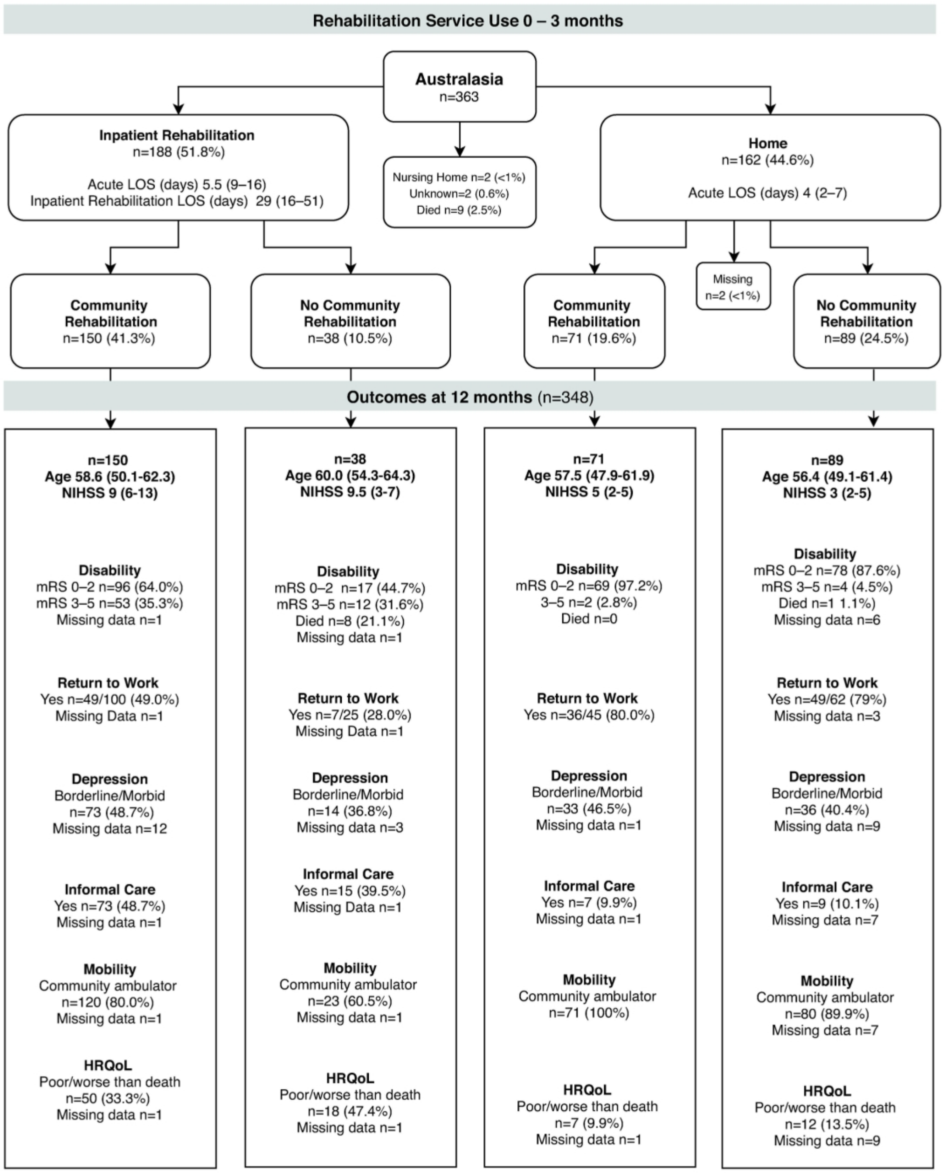

Figure 2 Rehabilitation service use and outcomes of participants ≤ 65 years in Australasia
Participants in IR at 3 months n=28 (7.7%); at 12months n=3 (<1%)
Participants receiving CR at 3 months n=1 (<1%), at 12 months n=39 (10.7%)
Participants who had not been discharged from their initial acute admission or were still in IR at the time of 3-month follow-up were allocated to the appropriate pathway using CR data collected at 12 months.
LOS (Length of Stay), Age, NIHSS data are median (IQR)
Depression (IDA scale), HRQoL (AQoL-4D), Community ambulator defined as Rivermead Motor Assessment Gross Function >9
12 month outcomes data are % of group, except Return to Work data which are % of those working prior to stroke
Missing 12 month outcomes data: Unknown Missing n=10/69 (14.5%); Alive Missing n=59/69 (85.5%)

173x213mm (300 x 300 DPI)

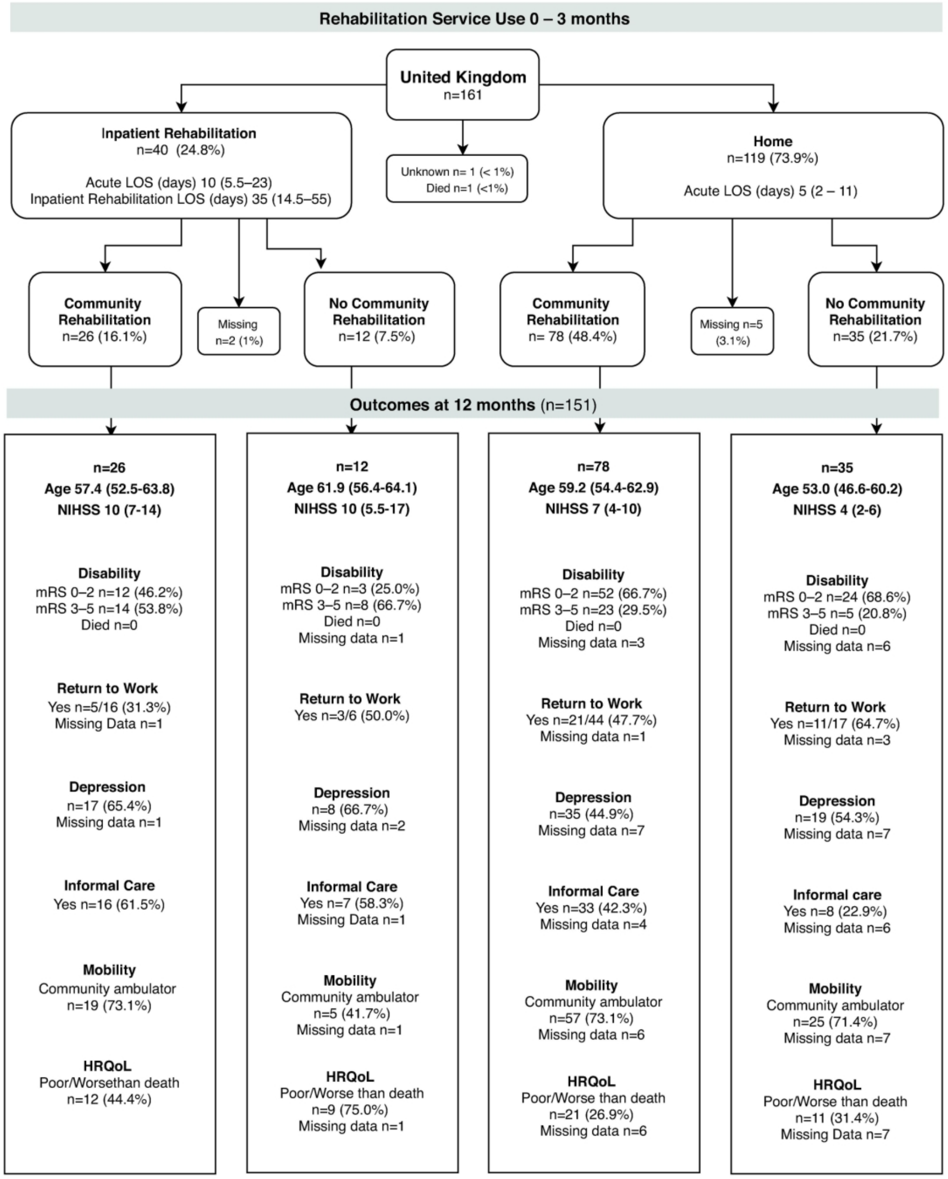

Figure 3 Rehabilitation service use and outcomes of participants ≤ 65 years in United Kingdom
Participants in inpatient rehabilitation at 3 months n=8 (5.0%); at 12 months n=1 (<1%)
Participants receiving community rehabilitation services at 3months n=1 (<1%), at 12 months n=11 (6.8%)
Participants who had not been discharged from their initial acute admission or were still in IR at the time of 3 month follow-up were allocated to the appropriate pathway using CR data collected at 12 months.
LOS (Length of Stay), Age, NIHSS data are median (IQR)
Depression (IDA scale), HRQoL (AQoL-4D), Community ambulator defined as Rivermead Motor Assessment Gross Function >9
12 month data are % of group, except Return to Work data which are % of participants in group working prior to stroke
Missing 12 month outcomes data: Unknown Missing n=15/71 (21.1%); Alive Missing n=56/71 (78.9%)

173x213mm (300 x 300 DPI)

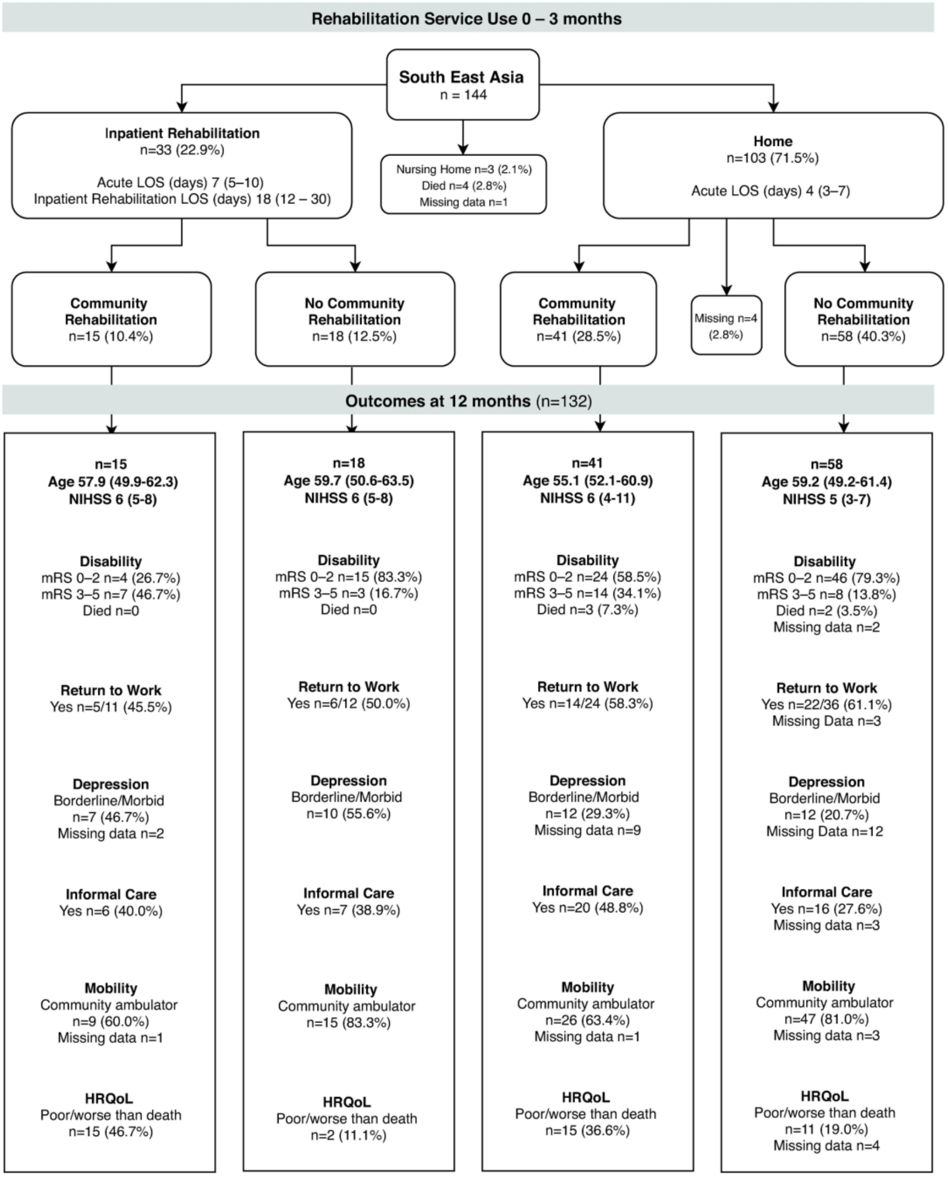

Figure 4 Rehabilitation service use and outcomes of participants ≤ 65 years in South East Asia
Participants in inpatient rehabilitation at 3 months n=0; at 12 months n=0
Participants receiving community rehabilitation services at 3 months n=2 (1.4%), at 12 months n=0
Participants who had not been discharged from their initial acute admission or were still in IR at the time of 3 month follow-up were allocated to the appropriate pathway using CR data collected at 12 months.
LOS (Length of Stay), Age, NIHSS data are median (IQR)
Depression (IDA scale), HRQoL (AQoL-4D), Community ambulator defined as Rivermead Gross Motor Function >9
12 month data are % of group, except Return to Work data which are % of participants in group working prior to stroke
Missing 12 month outcomes data: Unknown Missing n=6/40 (15%); Alive Missing n=34/40 (85%)

173x213mm (300 x 300 DPI)

Supplement 1

S-Table 1 Proportion of stroke survivors using different rehabilitation services in each region by two major prognostic factors: stroke severity and age

Region	Stroke Severity / Age	n	Inpatient Rehabilitation & Community Rehabilitation	Inpatient Rehabilitation & No Community Rehabilitation	Home & Community Rehabilitation	Home & No Rehabilitation Service Use
			n=150	n=38	n=71	n=89
Australasia	Mild	209	59 (28%)	12 (6%)	58 (28%)	80 (38%)
	Moderate	103	67 (65%)	15 (15%)	12 (12%)	9 (9%)
	Severe	36	24 (67%)	11 (31%)	1 (3%)	0 (0%)
	16-45	54	23 (43%)	1 (2%)	14 (26%)	16 (30%)
	46-65	294	127 (43%)	37 (13%)	57 (19%)	73 (25%)
			n=26	n=12	n=78	n=35
UK	Mild	87	8 (9%)	4 (5%)	45 (52%)	30 (34%)
	Moderate	53	15 (28%)	5 (9%)	28 (53%)	5 (9%)
	Severe	11	3 (27%)	3 (27%)	5 (45%)	0 (0%)
	16-45	17	2 (12%)	0 (0%)	7 (41%)	8 (47%)
	46-65	134	24 (18%)	12 (9%)	71 (53%)	27 (20%)
			n=15	n=18	n=41	n=58
SE Asia	Mild	93	11 (12%)	12 (13%)	24 (26%)	46 (49%)
	Moderate	34	4 (12%)	6 (18%)	14 (41%)	10 (29%)
	Severe	5	0 (0%)	0 (0%)	3 (60%)	2 (40%)
	16-45	21	2 (10%)	3 (14%)	5 (24%)	11 (52%)
	46-65	111	13 (12%)	15 (14%)	36 (32%)	47 (42%)

For peer review only

Supplement 2

S-Table 2. Recommendations for post stroke rehabilitation from Australia, UK and Singapore clinical guidelines during AVERT

Guideline	Recommendation
Australia (NSF, 2005 & 2007)[32,50]	All people admitted to hospital with stroke and who require rehabilitation should be treated in a comprehensive or rehabilitation stroke unit with an interdisciplinary team (Level I)<br><br>If ongoing inpatient rehabilitation is needed, care should be provided in either a stroke rehabilitation unit or a general rehabilitation unit (Grade A; Level I)<br><br>Rehabilitation in the community is equally effective if delivered in the hospital via outpatients, or day hospital, or in the community, and should be offered to all stroke patients as needed (Grade A; Level I)<br><br>Where comprehensive interdisciplinary community rehabilitation services and carer support services are available, early supported discharge services may be provided for people with mild to moderate disability (Level I)
UK (RCP 3rd edition, 2008)[51]	Once a patient is medically investigated, treated and stable, rehabilitation should be delivered by a specialist stroke service (in an inpatient unit, or a day-hospital unit, or at home)<br><br>All patients discharged home directly after acute treatment but with residual problems should be followed up by specialist stroke rehabilitation services<br><br>Domiciliary rehabilitation services should be commissioned as part of an 'early supported discharge' scheme to deliver specialist rehabilitation at home in liaison with inpatient services, as well as in the long-term<br><br>Patients should only be discharged early (before the end of acute rehabilitation) from National Clinical Guideline for Stroke hospital if there is a specialist stroke rehabilitation team able to continue rehabilitation in the community from the day of transfer and if the patient is able to transfer safely from bed to chair, and if other problems can be safely managed at home
SIngapore (MoH, 2009)[29]	Stroke patients should receive organized inpatient multidisciplinary rehabilitation (Grade A, Level 1+)<br><br>If able to do so, stroke patients should be encouraged to participate in more intensive rehabilitation particularly in the first six months (Grade A, Level 1+)

<u>Levels of evidence</u>
NSF (2005, 2007)[32,50]
Level I Evidence obtained from a systematic review of all relevant randomised controlled trials. Grade A recommendation: A Body of evidence can be trusted to guide practice

Singapore MoH (2009)[29]
Level 1+: Well conducted meta-analyses, systematic reviews of RCTs, or RCTs with a low risk of bias.
Grade A Recommendation: At least one meta-analysis, systematic review of RCTs, or RCT rated as 1+ + and directly applicable to the target population; or A body of evidence consisting principally of studies rated as 1+, directly applicable to the target population, and demonstrating overall consistency of results

For peer review only

Print Form

CONSORT CHECKLIST

Table. CONSORT 2010 Checklist of Information to Include When Reporting a Randomized Trial[a]

Section and Topic	Item No.	Checklist Item	Reported on Page No.
**Title and abstract**			
	1a	Identification as a randomized trial in the title	n/a
	1b	Structured summary of trial design, methods, results, and conclusions (for specific guidance see CONSORT for abstracts)	3-4
**Introduction**			
Background and objectives	2a	Scientific background and explanation of rationale	5-6
	2b	Specific objectives or hypotheses	6
**Methods**			
Trial design	3a	Description of trial design (such as parallel, factorial) including allocation ratio	6-7
	3b	Important changes to methods after trial commencement (such as eligibility criteria), with reasons	n/a
Participants	4a	Eligibility criteria for participants	6
	4b	Settings and locations where the data were collected	6
Interventions	5	The interventions for each group with sufficient details to allow replication, including how and when they were actually administered	6
Outcomes	6a	Completely defined prespecified primary and secondary outcome measures, including how and when they were assessed	n/a
	6b	Any changes to trial outcomes after the trial commenced, with reasons	n/a
Sample size	7a	How sample size was determined	6
	7b	When applicable, explanation of any interim analyses and stopping guidelines	6
Randomization Sequence generation	8a	Method used to generate the random allocation sequence	6
	8b	Type of randomization; details of any restriction (such as blocking and block size)	6
Allocation concealment mechanism	9	Mechanism used to implement the random allocation sequence (such as sequentially numbered containers), describing any steps taken to conceal the sequence until interventions were assigned	6
Implementation	10	Who generated the random allocation sequence, who enrolled participants, and who assigned participants to interventions	6
Blinding	11a	If done, who was blinded after assignment to interventions (for example, participants, care providers, those assessing outcomes) and how	6
	11b	If relevant, description of the similarity of interventions	6
Statistical methods	12a	Statistical methods used to compare groups for primary and secondary outcomes	9
	12b	Methods for additional analyses, such as subgroup analyses and adjusted analyses	9
**Results**			
Participant flow (a diagram is strongly recommended)	13a	For each group, the numbers of participants who were randomly assigned, received intended treatment, and were analyzed for the primary outcome	9-10
	13b	For each group, losses and exclusions after randomization, together with reasons	9-10
Recruitment	14a	Dates defining the periods of recruitment and follow-up	6,7,8
	14b	Why the trial ended or was stopped	6
Baseline data	15	A table showing baseline demographic and clinical characteristics for each group	9-10
Numbers analyzed	16	For each group, number of participants (denominator) included in each analysis and whether the analysis was by original assigned groups	n/a
Outcomes and estimation	17a	For each primary and secondary outcome, results for each group, and the estimated effect size and its precision (such as 95% confidence interval)	n/a
	17b	For binary outcomes, presentation of both absolute and relative effect sizes is recommended	n/a
Ancillary analyses	18	Results of any other analyses performed, including subgroup analyses and adjusted analyses, distinguishing prespecified from exploratory	8-12
Harms	19	All important harms or unintended effects in each group (for specific guidance see CONSORT for harms)	n/a
**Comment**			
Limitations	20	Trial limitations, addressing sources of potential bias, imprecision, and, if relevant, multiplicity of analyses	16
Generalizability	21	Generalizability (external validity, applicability) of the trial findings	16
Interpretation	22	Interpretation consistent with results, balancing benefits and harms, and considering other relevant evidence	12-16
**Other information**			
Registration	23	Registration number and name of trial registry	3
Protocol	24	Where the full trial protocol can be accessed, if available	ref 16
Funding	25	Sources of funding and other support (such as supply of drugs), role of funders	17

[a] We strongly recommend reading this statement in conjunction with the CONSORT 2010 Explanation and Elaboration for important clarifications on all the items. If relevant, we also recommend reading CONSORT extensions for cluster randomized trials, noninferiority and equivalence trials, nonpharmacological treatments, herbal interventions, and pragmatic trials. Additional extensions are forthcoming: for those and for up-to-date references relevant to this checklist, see http://www.consort-statement.org.

©2010 American Medical Association. All rights reserved.

For peer review only - http://bmjopen.bmj.com/site/about/guidelines.xhtml

BMJ Open

Exploring post-acute rehabilitation service use and outcomes for working age stroke survivors (≤65 years) in Australia, United Kingdom and South East Asia: Data from the international AVERT trial

Journal:	*BMJ Open*
Manuscript ID	bmjopen-2019-035850.R1
Article Type:	Original research
Date Submitted by the Author:	18-Mar-2020
Complete List of Authors:	Walters, Rosy; Royal Free London NHS Foundation Trust, Physiotherapy ; Florey Institute of Neuroscience and Mental Health - Austin Campus, Stroke<br>Collier, Janice M.; Florey Institute of Neuroscience and Mental Health - Austin Campus, Stroke<br>Braighi Carvalho, Lillian; Florey Institute of Neuroscience and Mental Health - Austin Campus, Stroke<br>Langhorne, Peter; University of Glasgow, Institute of Cardiovascular and Medical Sciences,<br>katijjahbe, Md Ali; Hospital Canselor Tuanku Muhriz, Physiotherapy; Swinburne University of Technology, Health Professions<br>Tan, Dawn; Singapore General Hospital, Department of Physiotherapy<br>Moodie, Marj; Deakin University, Deakin Health Economics<br>Bernhardt, Julie; Florey Institute of Neuroscience and Mental Health - Austin Campus, Stroke
**Primary Subject Heading**:	Neurology
Secondary Subject Heading:	Rehabilitation medicine
Keywords:	Stroke < NEUROLOGY, REHABILITATION MEDICINE, Organisation of health services < HEALTH SERVICES ADMINISTRATION & MANAGEMENT

SCHOLARONE™ Manuscripts

I, the Submitting Author has the right to grant and does grant on behalf of all authors of the Work (as defined in the below author licence), an exclusive licence and/or a non-exclusive licence for contributions from authors who are: i) UK Crown employees; ii) where BMJ has agreed a CC-BY licence shall apply, and/or iii) in accordance with the terms applicable for US Federal Government officers or employees acting as part of their official duties; on a worldwide, perpetual, irrevocable, royalty-free basis to BMJ Publishing Group Ltd ("BMJ") its licensees and where the relevant Journal is co-owned by BMJ to the co-owners of the Journal, to publish the Work in this journal and any other BMJ products and to exploit all rights, as set out in our licence.

The Submitting Author accepts and understands that any supply made under these terms is made by BMJ to the Submitting Author unless you are acting as an employee on behalf of your employer or a postgraduate student of an affiliated institution which is paying any applicable article publishing charge ("APC") for Open Access articles. Where the Submitting Author wishes to make the Work available on an Open Access basis (and intends to pay the relevant APC), the terms of reuse of such Open Access shall be governed by a Creative Commons licence – details of these licences and which Creative Commons licence will apply to this Work are set out in our licence referred to above.

Other than as permitted in any relevant BMJ Author's Self Archiving Policies, I confirm this Work has not been accepted for publication elsewhere, is not being considered for publication elsewhere and does not duplicate material already published. I confirm all authors consent to publication of this Work and authorise the granting of this licence.

For peer review only

Exploring post-acute rehabilitation service use and outcomes for working age stroke survivors(≤65 years) in Australia, United Kingdom and South East Asia: Data from the international AVERT trial

AUTHORS

Walters, R.[1,2]

Collier, J. M.[1]

BraighiCarvalho, L.[1]

Langhorne, P.[3]

Katijjahbe, M. A.[4,5]

Tan, D.[6]

Moodie, M.[7]

Bernhardt, J.[1]

on behalf of the AVERT Trialists' Collaboration

AFFILIATIONS

[1]Florey Institute of Neuroscience and Mental Health, University of Melbourne, Heidelberg Australia

[2]Physiotherapy, Royal Free London NHS Foundation Trust, London, United Kingdom

[3]Academic Section of Geriatric Medicine, Institute of Cardiovascular and Medical Sciences, University of Glasgow, Glasgow, United Kingdom

[4]Physiotherapy, Hospital CanselorTuankuMuhriz, PusatPerubatan UKM, Cheras Kuala Lumpur

[5]Department of Health Professions, Faculty of Health, Art and Design, Swinburne University of Technology, Hawthorn, Victoria.

[6]Physiotherapy, Singapore General Hospital, Singapore

[7]Deakin Health Economics, Deakin University, Burwood Australia

*Corresponding author: Julie Bernhardt, Florey Institute of Neuroscience and Mental Health – Austin Campus, Stroke, 245 Burgundy Street, Heidelberg, VIC, Australia 3084. Email: julie.bernhardt@florey.edu.auPh: 613 9305 7072

For peer review only

Word Count: 3739words

Tables n=1

Figures n=4

Supplementary Files n=2

Keywords: young stroke, rehabilitation services, return to work, outcome, international

For peer review only

ABSTRACT

Objectives Information about younger people of working age (≤65 years), theirpost-stroke outcomes and rehabilitation pathways can highlight areas for further research and service change. This paper describes:(i) baseline demographics; (ii) post-acute rehabilitation pathways; and (iii) 12 month outcomes; disability, mobility, depression, quality of life, informal care and return to work of working age people acrossthree geographical regions (Australasia (AUS), South East (SE) Asia, and United Kingdom (UK)).

DesignThis post hoc descriptive exploration of data from the large international very early rehabilitation trial (AVERT) examinedthe four common post-acute rehabilitation pathways (inpatient rehabilitation, home with community rehabilitation, inpatient rehabilitation then community rehabilitation and, home with no rehabilitation) experienced by participants in the 3 months post strokeand describestheir 12 month outcomes.

SettingHospital stroke units in AUS, UK and SE Asia.

Participants Acute stroke patients recruited within 24 hourswho were ≤65 years.

Results668 participants were ≤65 years; 99% lived independently, and 88% no disability (modified Rankin Score (mRS)=0) prior to stroke. We had complete data for 12 month outcomes for n=631 (94%). The proportion receiving inpatient rehabilitationwas higher in AUS than other regions (AUS 52%; UK 25%; SE Asia 23%), whereas the UK had higher community rehabilitation (UK 65%; AUS 61%; SE Asia 39%). At 12 months, 70% had no or little disability (mRS 0-2), 44% were depressed, 28% rated quality of life as poor or worse than death. For those working prior to stroke (n=228), only 57% had returned to work.A noteworthy number of working age survivors received no rehabilitation services within 3 monthspost stroke.

Conclusions There was considerable variation in rehabilitation pathways and post-acute service use across three regions. At 12 months there were high rates of depression, poor quality of life, and low rates of return to work.

Trial registration Australian New Zealand Clinical Trials Registry ACTRN12606000185561

For peer review only

Article Summary

Strengths and limitations of this study

- Large acute rehabilitation trial provides descriptive data and overview of regional service patterns.
- Little missing data provide high confidence in outcomes.
- Smaller numbers of participants and wide diversity of health services in SE Asia limit insights for this geographicalregion.

For peer review only

INTRODUCTION

Evidence suggests stroke incidence in people of working age is on the rise.[1]In Australia, New Zealand and the United Kingdom (UK), 25-30% of strokes occur in people of working age (≤65 years).[2-5] With 51,000 strokes/year in Australia[2] and 152,000/year in the UK[6], this equates to an estimated 12,750 and 38,000 strokes in people of working age each year in these countries respectively.

With five-year survival rates the highest for those 50 years and younger[7], stroke survivors of working age may live many years with the physical, psychological and social consequences of stroke. Stroke survivors of working age have unique rehabilitation needs and different expectations of recovery.[8-10] Outcomes pertinent to stroke survivors of working age may include a return to independence, self-reliance, and work.[11, 12] Alongside from the personal impact, the financial burden associated with stroke disability in people of working age, to individuals, carers and the economy is considerable. The cost of lost earnings due to reduced employment from stroke in Australia alone was estimated as $975 million in 2013.[2]Rehabilitation is a time limited, costly, but valued part of post-stroke care.

How stroke services are organised to meet the needs of survivors varies between countries. Service priorities and guideline implementation also vary across different healthcare settings, and according to government health structures, geography and culture.[13] Acute care in organised stroke units is supported by high level evidence, and global efforts to standardise acute medical treatments has improved stroke unit uptake, which typically includes some level of assessment and treatment by a multidisciplinary team. Inter-country or regional differences in rehabilitation access and pathways following acute care are less well understood and therefore afocus of this study.

Post-acute stroke rehabilitation services often include two key models:

- Inpatient Rehabilitation (IR): rehabilitation service delivered in the hospital setting
- Community Rehabilitation (CR): rehabilitation service delivered as an outpatient at a clinic or day hospital or in the patient's own home. This model includes Early Supported Discharge (ESD) which offers early discharge from acute hospital and provision of rehabilitation and support in the home.

For peer review only

The AVERT trial of very early rehabilitation[14] included 2104 participants from 5 countries (3 geographical regions; Australasia, South East Asia, United Kingdom) who were recruited within 24 hours of stroke, and randomised to two rehabilitation interventions: very early intensive mobilisation plus usual care, or usual care alone.Twelve month disability and health related quality of life outcomes were not different between intervention groups.[15] This large dataset provided a unique opportunity to explore rehabilitation pathways and 12 month outcomes for the subgroup of participants of working age.

Specifically, our aims were:

(i) To describe the demographics of working age stroke population in three regions

(ii) To explore the distribution of these individuals across four post-acute rehabilitation pathways within 3 monthspost stroke in three geographical regions

(iii) To describe 12 month outcomes: disability, mobility, depression, quality of life, informal care and return to work (RTW) in three regions

For peer review only

METHODS

We have defined 'working age stroke' as those aged 18-65 years who are considered to be of working age; as pension age in the UK, Australia & New Zealand is 65-67 years and minimum retirement in Malaysia and Singapore age is 60-62.[16]

Design

This is a post-hoc exploratory descriptive evaluation using data from the AVERT trial. AVERT was a single-blind, randomised controlled trial with a comprehensive economic evaluation in which participants were recruited from 56 acute stroke units between 2006 and 2015. Eligible participants for AVERT were aged 18 years or older, with no significant pre-morbid disability (modified Rankin Score ≤2), with confirmed first or recurrent stroke, admitted to a stroke unit within 24 hours of stroke onset, and medically stable. The AVERT trial method has been described in detail previously[14] with the trial protocol published.[17] Human Research Ethics Committee approval was obtained from all sites and conformed to the Declaration of Helsinki. Participants or representatives provided individual consent. We extracted patient demographics, baseline stroke characteristics, discharge location, rehabilitation care, and patient outcomes for participants ≤ 65 years and grouped these into the three geographical regions with similar health and social organisation: Australasia (Australia and New Zealand), United Kingdom (UK) (England, Scotland, Wales, Northern Ireland) and South East (SE) Asia (Singapore and Malaysia).

Patient and public involvement

A stroke consumer provided input throughout the trial planning and protocol development, site training, trial progress as a member of the trial management and executive committees, andcommunication of results.No public involved.

Demographics and baseline stroke characteristics

Demographics included age, sex, presence of comorbid vascular risk factors, previous stroke, pre-morbid disability (modified Rankin Scale, mRS)[18]walking independence and living arrangements prior to stroke. Baseline stroke characteristics included stroke severity (National Institute of Health Score (NIHSS) score: mild (NIHSS 1–7), moderate (8–16), and severe (>16)[19] and stroke type

For peer review only - http://bmjopen.bmj.com/site/about/guidelines.xhtml

(Oxfordshire Community Stroke Type Project classification).[20]Baseline walking used the gait component of the Mobility Scale for Acute Stroke[21] which measures the amount of assistance and independence with walking 10m indoors with or without gait aid using a 6-point scale ranging from 1 (unable) to 6 (independent).

Post-acute discharge pathways

Discharge to home and rehabilitation (inpatient and community) data were collected at both 3 and 12 months by face-to-face interview (blinded assessor) with participants and/or carers, with supporting documentation sourced from health care providers. Discharge to supported care (nursing home, supported accommodation) was not included as small numbers were expected. Our focus was on determining resources used, i.e., location of care (hospital, community, home) and days of rehabilitation, rather than on the content of the therapy itself. Information about rehabilitation services collected at the 3 month follow-up were used to classify participants into four different rehabilitation pathways.(Figure 1).

<Insert Figure 1 about here>

12 month outcomes

12 month outcomes are reported for the four post-acute discharge pathways. We selected 12 month outcomes which included measures aligned withthe International Classification of Functioning Disability and Health Framework.[22]

Functioning: The modified Rankin Scale (mRS)[18] is an ordinal scale ranging from 0 (no disability) to 5 (severe disability) or 6 (death). The proportionwith favourable outcome defined as mRS scores 0-2 (no or minimal disability), is reported here.

Activity: We used the Rivermead Motor Assessment Gross Function tool (a 13-point scale[23]) reliable in stroke[24] to measure community mobility at 3 and 12 months. We report the proportion achieving a score of 10-13 at 12 months, which indicates an ability to walk independently outside the home.[25]

Participation: Of those working prior to stroke, we report the proportion of stroke survivors who returned to work at 12 months in any capacity (part time or full time). Quality of life is reported using the Assessment of Quality of Life measure(AQoL-

For peer review only

4D). Thequestionnairecomprises 15 items over 5 subscales: independent living, social relationships, physical senses, psychological well-being, and illness.[26] TheAQoL utility score, which refers to the value people place on their Health Related Quality of Life (HRQoL), ranges from −0.04 (state worse than death), death (0.0) through to 1.0 (excellent).[27] We report the proportion of stroke survivors scoring *poor*or *worse than death* (score -0.04-0.4) at 12 months.

Body Function: We extracted depression data from the Irritability Depression and Anxiety (IDA) scale and report the proportion with 'Borderline' (score 4-6) or 'Morbid' depression (score >7)[28]at 12 months.

Environmental Factors: We reportthe proportion of stroke survivors requiring informal care at 12 months; defined as the participant requiring help with activities of daily living beyond that provided by formal support services.

Analyses

Baseline, rehabilitation service use within 3 months and 12month outcome data arepresented using descriptive analysis (n, %) using STATA IC for all analyses. Median and interquartile ranges are presented for age, baseline stroke severity (NIHSS score) and length of stay. We describe pathway use by baseline stroke severity (mild, moderate and severe), and by younger (18-45 years) and older (46-65) age. We elected not to conduct formal statistical comparisons given the exploratory nature of the study, the expected small sample sizes of subgroups across the rehabilitation pathways in the study and,most importantly, we had no a priori hypothesesabout the relationship between rehabilitation pathways and outcomes (or any other factors) that we felt could be formally tested in a robust way in this dataset given these expected limitations.

RESULTS

In the AVERT trial, 668 participants were ≤65 at time of stroke; 363 participants were from Australasia, 161 from the UK and 144 from the SE Asia region. Characteristics of participants are presented in Table 1. The median age of participants was similar across the three regions (58 years) with a high proportion of men (73%). Prior to stroke, 99% of participants were living independently, 88% had no disability

For peer review only

(mRS=0), 97% were walking without a gait aid and 62% were working. The highest proportion of participants with hypertension, diabetes mellitus and ischaemic heart disease were from SE Asia. Nearly two thirds of the people in SE Asia had lacunar strokes. The UK had the highest proportion of current smokers.

Table 1: Premorbid & baseline stroke characteristics of participants ≤65 by region

	Australasia n = 363	UK n=161	SE Asia n = 144	Total n = 668
**Baseline Characteristics**				
**Age**, **years** median (IQR)	57.6 (49.5-62.3)	58.4 (51.5-62.6)	57.6 (51.8-61.9)	57.9 (50.3-62.2)
range	19.93-65.99	37.53-65.98	16.4-65.54	16.4-65.99
**Sex, n (%)**				
Female	98 (27.0)	56 (34.8)	50 (34.7)	204 (30.5)
Male	265 (73.0)	105 (65.2)	94 (65.3)	464 (69.5)
**Risk factors, n (%)**				
Hypertension	198 (54.5)	84 (52.2)	105 (72.9)	387 (57.9)
Hypercholesterolemia	119 (32.8)	67 (41.6)	54 (37.5)	240 (35.9)
Diabetes mellitus	68 (18.7)	31 (19.3)	61 (42.4)	160 (24.0)
Ischemic heart disease	58 (16.0)	20 (12.4)	24 (16.7)	102 (15.3)
Atrial fibrillation	36 (9.9)	11 (6.8)	11 (7.6)	58 (8.7)
**Smoking, n (%)**				
Never Smoked	139 (38.3)	51 (31.7)	78 (54.2)	268 (40.1)
Smoker*	129 (35.5)	70 (43.5)	49 (34.0)	248 (37.1)
Ex-Smoker*	91 (25.1)	39 (24.2)	16 (11.1)	146 (21.)
Unknown	4 (1.1)	1 (<1)	1 (<1)	6 (<1)
**Premorbid data**				
**modified Rankin Scale, n (%)**				
0	319 (87.9)	133 (82.6)	135 (93.8)	587 (87.9)
1	26 (7.2)	17 (10.6)	5 (3.5)	48 (7.2)
2	18 (5.0)	11 (6.8)	4 (2.8)	33 (4.9)
**Independent walking noaid, n (%)**	356 (98.1)	151 (93.8)	139 (96.5)	646 (96.7)
**Living arrangement at time of admission, n (%)**				
Home alone	52 (14.3)	38 (23.6)	22 (15.3)	112 (16.8)
Home with someone	310 (85.4)	122 (75.8)	121 (84.0)	553 (82.8)
Supported accommodation	1 (<1)	0 (0)	1 (<1)	2 (<1)
Other	0 (0)	1 (<1)	0 (0)	1 (<1)
**Employment Status, n (%)**				
Working at time of stroke	238 (65.6)	89 (55.3)	86 (59.7)	413 (61.8)
Full Time	188 (79.0)	66 (74.2)	73 (84.9)	327 (79.2)

For peer review only

Part Time	50 (21.0)	23 (25.8)	13 (15.1)	86 (20.8)
**Baseline stroke**				
**First stroke, n (%)**	315 (86.8)	141 (87.6)	112 (77.8)	568 (85.0)
NIHSS score, median (IQR)	6 (4-11)	6 (4-11)	5 (4-8)	6 (4-10)
Mild (1-7)	213 (58.7)	93 (57.8)	99 (68.8)	405 (60.6)
Moderate (8-16)	107 (29.5)	56 (34.8)	39 (27.1)	202 (30.2)
Severe (>16)	43 (11.9)	12 (7.5)	6 (4.2)	61 (9.1)
**Stroke type, n (%)**				
Oxfordshire Stroke Classification				
Total anterior circulation infarct	61 (16.8)	28 (17.4)	8 (5.6)	97 (14.5)
Partial anterior circulation infarct	109 (30.0)	55 (34.2)	21 (14.6)	185 (27.7)
Posterior circulation infarct	53 (14.6)	11 (6.8)	7 (4.9)	71 (10.6)
Lacunar infarct	80 (22.0)	50 (31.1)	91 (63.2)	221 (33.1)
Haemorrhage				
Intracerebral haemorrhage	60 (16.5)	17 (10.6)	17 (11.8)	94 (14.1)
**Baseline walking, n (%)** (Mobility Scale for Acute Stroke walking score)				
Independent	45 (12.4)	31 (19.3)	12 (8.3)	88 (13.2)
Supervised or assisted	200 (55.1)	61 (37.9)	83 (57.6)	344 (51.5)
Unable to walk	117 (32.2)	69 (42.9)	49 (34.0)	235 (35.2)
Missing or unknown	1 (<1)	0 (0)	0 (0)	1 (<1)

NIHSS=National Institute of Health Stroke Scale;IQR=Interquartile Range
*Smoker is defined as a current smoker or participant who had quit smoking in the past 2 years, and an ex-smoker as a participant who had quit smoking more than 2 years ago

For peer review only

Rehabilitation Pathways

The pathways of rehabilitation service use to 3 months, and 12 month outcome summaries for each region are shown in Figures 2-4. Of the 668 participants, 94% (n=631/668) followed one of the four common pathways of post-acute discharge. Most were discharged home (n=384/668, 57%) or to inpatient rehabilitation (n=261/668, 39%). Fourteen participants died in acute care. The discharge destination of the remaining participants was a nursing home (n=5), with missing discharge (n=4) and community rehabilitation (n=14) data.

<Insert Figure 2 about here>

<Insert Figure 3 about here>

<Insert Figure 4 about here>

In the first 3 months post stroke, 67% of participants received rehabilitation (IR or CR) services. Only 8% were still receiving rehabilitation services at 12 month follow up. The proportion receiving IR was higher in Australasia than other regions (Australasia 52%; UK 25%; SE Asia 23%). The length of stay in IR was longest in UK (median 35 days) and shortest in SE Asia (median 18 days) and the UK had higher participation in CR than other regions (UK 65%; Australasia 61%; SE Asia 39%). It was most common for UK stroke survivors to go home from acute care and receive CR (48%). For those discharged home from acute care, the length of stay in hospital was similar in the three regions (median 4-5 days). The number of working age survivors who went home after a short acute care stay and received no rehabilitation (IR or CR) was unexpectedly high (Australasia 25%; UK 22%; SE Asia 40%).

The proportion of stroke survivors with mild, moderate and severe stroke, grouped by younger versus older age, and their service use pathways by regions is shown in Supplement 1. As could be anticipated, the highest proportion of working age survivors who did not receive rehabilitation were those with mild stroke (40%, n=156/389) compared to those with moderate (12%, n=24/200) or severe (4%, n=2/52) stroke. Less expected was our finding that 38% (n=35/92) of the younger age group aged 18-45 years did not receive any rehabilitation on discharge from acute care.

12 month outcomes

Of the 631 working age stroke survivors included in the four discharge pathways, 2% (n=14/631) had died by 12 month follow up. Some 70% (n=444/631) had a favourable outcome (mRS 0-2) reporting little or no disability (mRS missing data 3%, n=20/631), yet 34% (n=217/631) were still receiving informal care. 79% (n=497/631) were community ambulators (Rivermead Mobility>9) but only 57% of those working prior to stroke (n=228/398) had returned to work. The proportion who had returned to work at 12 months was similar across the three regions: Australasia 61%, SE Asia 57%, UK 48%. 28% (n=174/631) had a HRQoL rated as poor or worse than death: UK 34%, SE Asia 27%, and Australasia 25%. Borderline or morbid depression was reported by 44% (n=276/631), with the highest proportion in the UK 52% and the lowest proportion in SE Asia 31%. The largest proportion of missing data was for

For peer review only

depression as some stroke survivors were unable to complete the questionnaire (IDA, 10%, n=65/631).

DISCUSSION

This report of rehabilitation service use and 12 month outcomes of working age stroke survivors across three geographical regions with differing health services using this well characterised and large dataset has yielded some unique findings. Interestingly, while countries in all three regions in this study have universal healthcare as a core principle of their healthcare system, we saw considerable variation in rehabilitation pathways and service use across the regions. For example, around 50% of working age stroke survivors in Australasia received inpatient rehabilitation, compared with around 25% of stroke survivors in the UK or SE Asia. In the case of SE Asia, many went straight home from acute stroke care and 40% of these individuals had no further rehabilitation. Whether this reflects differences in access to inpatient rehabilitation services between these regions, or other factors, was not examined in this study. The SE Asia data from AVERT included data from Singapore and Malaysia. Singapore is classified as an 'advanced economy' and Malaysia as a 'developing economy'.[29] The MalaysianClinical Practice Guidelines 2012[30]do not address care beyond the acute stroke unit. Indeed, all working age stroke survivorsin SE Asia admitted to inpatient rehabilitation (n=33) were from Singapore. It should be noted that Singapore stroke guidelines[31] recommend that 'stroke patients should receive organised inpatient multidisciplinary rehabilitation' (Supplement 2). In this dataset, only 51% (n=33/65) of participants from Singapore received inpatient rehabilitation.

The larger proportion of working age stroke survivors from SE Asia who went home with no rehabilitation compared to those in Australasia or UK may be partly explained by a difference in stroke severity. However, median baseline stroke severity for this group was similar across the three regions (median NIHSS 5 SE Asia; 3 AUS; 4 UK). It may reflect the access to community rehabilitation services in the region. There is currently no protocol in Malaysia which specifically addresses the transfer of care for long-term post stroke management at community level.[32] A recent study of post stroke management in Malaysia[32] found that almost one third of

For peer review only - http://bmjopen.bmj.com/site/about/guidelines.xhtml

patients (31%) were not referred to any rehabilitation facility. They cited the lack of awareness among physicians regarding the role of neuro rehabilitation and the lack of co-ordination of post stroke care beyond the acute phase as contributing factors. It is possible that participants were offered a referral for outpatient rehabilitation services but declined due to the cost incurred, or that the group included overseas participants who were not eligible for local rehabilitation services.

In contrast with SE Asia, the lower rates of inpatient rehabilitation in the UK appear to be offset by the higher use of community rehabilitation, with 48% of UK working age stroke survivors discharged home from acute care with community rehabilitation. Early supported discharge (ESD) services have been a focus of service development in the UK over a number of years. The ESD model has been shown to reduce length of stay and long-term dependency at 12 months in people with mild to moderate stroke severity.[33] We found that the majority of stroke survivors who received community rehabilitation were those with mild and moderate stroke, consistent with guidelinecriteria for an ESD service.[34] Only 20% of survivors in Australasia went directly home with community rehabilitation. Although ESD has been recommended in the Australian Clinical Practice Guidelines[35, 36]since 2005 (Supplement 2), if we assume that episodes of community rehabilitation reflect ESD, so far this model does not appear to be common practice in Australia. A challenge with interpreting community rehabilitation data is that it is often poorly described, and the recommended comprehensive ESD may not be implemented.

We were surprised to find that nearly a quarter of stroke survivors in the UK and Australasia had no rehabilitation services in the first three months post stroke. None of these individuals had severe stroke, so perceived absence of 'rehabilitation potential' would not explain this finding. In the UK and Australasia, a large proportion (30-47%) of the younger age group 18-45, went home with no rehabilitation.The same pattern was present for those with mild stroke at baseline, with 34-38% of these individuals in UK and Australasia not receiving rehabilitation. This finding may relate to professionals' expectations that younger survivors and those with mild stroke will spontaneously recover without services. The need for rehabilitation

For peer review only

services may not have been identified; ‘mild’ symptoms may not be apparent in the acute setting, and some, such as mood disturbance, may have a delayed onset. Stroke is a sudden, life-changing event for a young adult, the effects of which may not be anticipated during acute care. A systematic review of qualitative research reports a young adult stroke describing; ‘When you’re in hospital, you don’t really think about how your life is going to be when you leave. You think that you’ll just carry on as before, but you don’t. You get home and that’s when the trauma starts’.[37]A flexible model of stroke service use and follow-up is indicated, such as the ‘point of contact with specialist services’ recommended in Parkinson’s Disease,[3] to provide ongoing support for working age stroke.

Across all three regions, we found that 12 month outcomes for those who did not receive rehabilitation were less than optimal. Whether an episode of rehabilitation would have improved these outcomes is unknown. What is perhaps most pertinent is whether the rehabilitation needs of patients in the acute phase of care were formally assessed. This was not a focus of the AVERT trial. Advances in post stroke interventions, such as thrombolysis and endovascular clot retrieval, have led to ongoing reductions in acute length of stay post-stroke.[38, 39] Less time in hospital may limit time for multidisciplinary assessment and identification of post-discharge needs. With a median acute length of stay for participants discharged directly home of 4-5 days in each of the three regions, it may be difficult for rehabilitation needs to be adequately assessed.The need for standardised assessment of patients in acute care for stroke rehabilitation has led to the development of Assessment for Rehabilitation: Pathway and Decision-Making Tool.[40]It is now recommended that all stroke survivors who are not for palliative care should be considered for rehabilitation.[40]Our data suggests that for a significant number of working age stroke survivors, the acute care stay may be their only contact with specialised medical and allied health stroke services, and that those under 45 years and those with mild stroke may be vulnerable to a lack of coordinated specialist post discharge care.

In this study, 70% reportedlittle to no disability at 12 months(favourable outcome mRS 0-2), which is consistent with previous research of those with working age stroke.[41-43]A large number of working age stroke survivors reported low mood (44% with borderline or morbid depression) and poor quality of life (28% rated HRQoL as

For peer review only

poor or worse than death; missing data n=30). Only 7% of healthy people the same age report this level of HRQoL,[27] suggesting the need for research to determine if targeted interventions can improve QoL in working age stroke survivors. Depression has been shown to be independently associated with low HRQoL in working age stroke survivors[44] and may be improved with psychosocial, neuropsychological or pharmacological interventions.[5]The Australian Stroke Guidelines[45] recommend routine assessment of mood disturbance post stroke, and the UK[5] has identified that service improvements in the organisation and delivery of psychological services for stroke survivors are needed. The psychosocial needs of working age stroke survivors are commonly reported as being 'unmet'[46-48]and surveillance and rehabilitation programs that specifically address these issues appear needed.

The proportion of working age stroke survivors (57%) in this study who had returned to work at 12 months was low but consistent with previous research.[49, 50]Poor rates of return to work despite good functional outcomes have been reported. For example, Varona and colleagues[43]evaluated long-term outcomes in younger adults after first ischemic stroke and found while 90% were independent, only 53% had returned to work. Return to work post stroke is complex and multifactorial[51, 52], with optimal interventions to improve return to work outcomes for people of working age being poorly understood.[53]A recent systematic review[50]concluded that either specialised vocational rehabilitation, conventional stroke rehabilitation or their combination is needed to increase return-to-work rates and improve the quality of life for stroke survivors of working age.

Study limitations

We elected to explore data for all stroke survivors of 'working age' (i.e ≤65 years) which is consistent with other working age definitions. However, in our study population, only 62% of participants were in formal paid employment prior to their stroke. We acknowledge that some people continue to work after age 65, and that the absence of information about unpaid, productive work is a limitation of the data. Further, the absence of information about the specific goals or types of rehabilitation has limited our ability to describe in more detail the rehabilitation services on offer.We relied on clinicians, patients and families reporting of rehabilitation services, and did not gather detailed information as part of the trial about services (such

For peer review only

asprofessions involved, details of service models) that could help benchmark services to any common standard. Such information would be valuable in future research.Smaller numbers of participants and wide diversity of health services in SE Asia limit insights for this region. Nevertheless, the data available from three separate geographical regions offer unique insights into who gets into rehabilitation, and what type, but not why. We elected not to run exploratory analyses on these data given the very small numbers of patients in sub-groups of interest, such as those of younger age (<45 years), different genders, or with mild stroke. Finally, younger stroke survivors live many years with post stroke disability and their needs may change over time. This study reflects only the first 12 months after stroke.

Conclusions

Despite the majority of working age stroke survivors in this study receiving rehabilitation services within the first three months, many reported low mood, poor QoL and failure to return to work at 12 months. Over a quarter (28%) of working age stroke survivors received no rehabilitation services. Younger individuals and those with mild stroke may not be offered rehabilitation yet report sub-optimal outcomes in the longer term. At present, there is little research targeting the needs of younger survivors or indicating benefit, this is an avenue for further enquiry.Further research into the most effective and cost-effective rehabilitation service use pathway for young adults with varying severity of stroke is needed to assist in the development of specific evidence-based guidelines to inform service delivery.

Acknowledgements

We thank the AVERT Collaboration investigators for their hard work and dedication, and the participants in this trial and their supporters. We also thank Brooke Parsons, Stroke Consumer, for her important and sustained contribution to this study. The Florey Institute of Neuroscience and Mental Health acknowledges the support received from the Victorian Government via the Operational Infrastructure Support Scheme.

Collaborators

The AVERT Trialists' Collaboration[15]

Author Contributions

For peer review only

RW and JB conceived the study and all authors contributed to the study design. Rehabilitation services data was devised by MM, data was managed by JMC and collected by the AVERT Trialists' Collaboration. RW performed data analysis. Interpretation was provided by JB, JMC, LBC, PL, KMA and DT. RW was the primary writer of the manuscript, with revisions and intellectual content from all authors. All authors provided final approval of the version to be published.

Funding

The AVERT trial was initially supported by the National Health and Medical Research Council (NHMRC) of Australia (grant numbers 386201 and 1041401). Additional funding was received from Chest Heart and Stroke Scotland (Res08/A114), Northern Ireland Chest Heart and Stroke, Singapore Health (SHF/FG401P/2008), the UK Stroke Association (TSA2009/09), and the UK National Institute of Health Research (HTA Project 12/01/16). NHMRC fellowship funding was provided to JB (1058635, 1154904). JB also received fellowship funding from the Australia Research Council (0991086) and the National Heart Foundation. RW received a Clinical Stipend from the NHMRC Centre of Research Excellence in Stroke Rehabilitation and Brain Recovery (1077898).

Competing interests

Ms Walters reports a clinical stipend from a NHMRC grant. Dr. Langhorne reports grants from NIHR, Stroke Association, Chest Heart and Stroke Scotland. Dr Bernhardt reports grants from the NHMRC, Stroke Association, Chest Heart and Stroke Scotland, Northern Ireland Chest Heart and Stroke, NIHR, ARC and NHF. Dr Moodie reports grants from NHMRC. Drs Tan and Bernhardt reports grants from Singapore Health. Drs Collier, BraighiCarvalho and Katijjahbe report nothing to disclose.

Patient consent for publication

Consent was obtained for de-identified publication of group data.

Data sharing statement

Data may be shared by writing to the corresponding author to obtain details of procedures and processes.

For peer review only

Open access

This is an open access article distributed in accordance with the Creative Commons Attribution Non Commercial (CC BY-NC 4.0) license, which permits others to distribute, remix, adapt, build upon this work non-commercially, and license their derivative works on different terms, provided the original work is properly cited, appropriate credit is given, any changes made indicated, and the use is non-commercial. See: http:// creativecommons. org/ licenses/ by- nc/ 4. 0/.

References

1. Béjot Y, Delpont B, Giroud M. Rising stroke incidence in young adults: More epidemiological evidence, more questions to be answered. *JAHA. 2016; 5*: e003661. doi:10.1161/JAHA.116.003661
2. Deloitte Access Economics. The economic impact of stroke in Australia. 2013
3. National Institute for Health and Care Excellence. NICE Guidance. Parkinson's Disease. 2018
4. Tobias M, Cheung J, Carter K, Anderson C, Feigin VL. Stroke surveillance: population based estimates and projections for New Zealand. *Aust. N. Z. J. Public Health*. 2007;31:520-525. doi: 10.1111/j.1753-6405.2007.00136.x
5. Royal College of Physicians. National clinical guideline for stroke 2016. Prepared by the Intercollegiate Stroke Working Party. 2016;Fifth Edition
6. Townsend N, Wickramasinghe K, Bhatnagar P, Smolina K, Nichols M, Leal J, Luengo-Fernandez R, Rayner M. Coronary heart disease statistics 2012. 2012
7. Smajlovic D, Kojic B, Sinanovic O. Five-year survival-after first-ever stroke. *Bosn. J. Basic Med. Sci.* 2006;6:17-22. doi: 10.17305/bjbms.2006.3138
8. Morris R. The psychology of stroke in young adults: The roles of service provision and return to work. *Stroke Res Treatment*. 2011:534812. doi: 10.4061/2011/534812
9. Dale Stone S. Reactions to invisible disability: the experiences of young women survivors of hemorrhagic stroke. *Disabil. Rehabil.* 2005;27:293-304. doi: 10.1080/09638280400008990
10. Teasell RW, McRae MP, Finestone HM. Social issues in the rehabilitation of younger stroke patients. *Arch. Phys. Med. Rehabil.* 2000;81:205-209. doi: 10.1016/s0003-9993(00)90142-4
11. Dixon G, Thornton EW, Young CA. Perceptions of self-efficacy and rehabilitation among neurologically disabled adults. *Clinical Rehabilitation.* 2007;21:230-240. doi: 10.1177/0269215506071784
12. Lawrence M, Kinn S. Needs, priorities, and desired rehabilitation outcomes of family members of young adults who have had a stroke: findings from a phenomenological study. *Disabil. Rehabil.* 2013;35:586-595. doi: 10.3109/09638288.2012.711895
13. Putman K, De Wit L. European comparison of stroke rehabilitation. *Top. Stroke Rehabil.* 2009;16:20-26. doi: 10.1310/tsr1601-20
14. The AVERT Trial Collaboration group, Bernhardt J, Langhorne P, Lindley RI, Thrift AG, Ellery F, Collier J, Churilov L, Moodie M, Dewey H, Donnan G.

For peer review only

Efficacy and safety of very early mobilisation within 24 h of stroke onset (AVERT): a randomised controlled trial. *The Lancet*. 2015;386:46-55. doi: 10.1016/S0140-6736(15)60690-0
15. Cumming TB, Churilov L, Collier J, Donnan G, Ellery F, Dewey H, Langhorne P, Lindley RI, Moodie M, Thrift AG, Bernhardt J, on behalf of the AVERT Trial Collaboration Group. Early mobilization and quality of life after stroke. Findings from AVERT. *Neurology*. 2019;93:e1-e12. doi: 10.1212/WNL.0000000000007937
16. The Statutes of the Republic of Singapore. Retirement and Re-Employment Act (chapter 274A). Revised Edition 2012:1-30
17. Langhorne P, Wu O, Rodgers H, Ashburn A, Bernhardt J. A Very Early Rehabilitation Trial after stroke (AVERT): a phase III, multicentre, randomised controlled trial. *Health Technol. Assess.* 2017;21:1-120. doi: 10.3310/hta21540
18. Van Swieten J, Koudstaal P, Visser M, Schouten H, Van Gijn J. Interobserver agreement for the assessment of handicap in stroke patients. *Stroke*. 1988;19:604-607. doi: 10.1161/01.str.19.5.604
19. Briggs DE, Felberg RA, Malkoff MD, Bratina P, Grotta JC. Should mild or moderate stroke patients be admitted to an intensive care unit? *Stroke*. 2001;32:871-876. doi: 10.1161/01.str.32.4.871
20. Bamford J, Sandercock P, Dennis M, Warlow C, Burn J. Classification and natural history of clinically identifiable subtypes of cerebral infarction. *The Lancet*. 1991;337:1521-1526. doi: 10.1016/0140-6736(91)93206-o
21. Simondson JA, Goldie P, Greenwood KM. The Mobility Scale for Acute Stroke Patients: concurrent validity. *Clin. Rehabil.* 2003;17:558-564. doi: 10.1191/0269215503cr650oa
22. World Health Organization. *International Classification of Functioning, Disability and Health: ICF*. World Health Organization; 2001.
23. Lincoln N, Leadbitter D. Assessment of motor function in stroke patients. *Physiotherapy*. 1979;65:48-52.
24. Adams S, Pickering R, Ashburn A, Lincoln N. The scalability of the Rivermead Motor Assessment in non-acute stroke patients. *Clin. Rehabil.* 1997;11:52-59. doi: 10.1177/026921559701100108
25. Cumming TB, Thrift AG, Collier JM, Churilov L, Dewey HM, Donnan GA, Bernhardt J. Very early mobilization after stroke fast-tracks return to walking: further results from the phase II AVERT randomized controlled trial. *Stroke*. 2011;42:153-158.
26. Hawthorne G, Richardson J, Osborne R. The Assessment of Quality of Life (AQoL) instrument: a psychometric measure of health-related quality of life. *Qual. Life Res.* 1999;8:209-224. doi: 10.1023/a:1008815005736
27. Hawthorne G, Osborne R. Population norms and meaningful differences for the Assessment of Quality of Life (AQoL) measure. *Aust. N. Z. J. Public Health*. 2005;29:136-142. doi: 10.1111/j.1467-842x.2005.tb00063.x
28. Snaith RP, Constantopoulos AA, Jardine MY, McGuffin P. A clinical scale forthe self-assessment of irritability. *Br. J. Psychiatry*. 1978;132:164-171. doi: 10.1192/bjp.132.2.164
29. International Monetary Fund. (2015). *World Economic Outlook: Uneven Growth—Short- and Long-Term Factors*. 2015. Washington (April)

For peer review only

30. Malaysian Society of Neurosciences, Academy of Medicine Malyasia, Ministry of Health Malaysia. Clinical Practice Guidelines. Management of Ischaemic Stroke. 2nd Edition 2012:1-64
31. Ministry of Health. Stroke and Transient Ischaemic Attacks Assessment, Investigation, Immediate Management and Secondary Prevention. *MOH Clinical Practice Guidelines 2/2009*. 2009
32. Abdul Aziz AF, Ali MF, Yusof MF, Che' Man Z, Sulong S, Aljunid SM. Profile and outcome of post stroke patients managed at selected public primary care health centres in Peninsular Malaysia: A retrospective observational study. *Sci. Rep.* 2018;8:17965. doi: 10.1038/s41598-018-36154-0
33. Langhorne P, Baylan S, Early Supported Discharge Trialists. Early supported discharge services for people with acute stroke. *Cochrane Database Syst. Rev.* 2017;7:CD000443. doi: 10.1002/14651858.CD000443.pub4
34. Intercollegiate Stroke Working Party. *National Clinical Guidelines for Stroke (Third Edition)*. London: Royal College of Physicians; 2008
35. National Stroke Foundation. Clinical Guidelines for Stroke Rehabilitation and Recovery. 2005
36. National Stroke Foundation. *Clinical guidelines for acute stroke management*. Melbourne 2007.
37. Lawrence M. Young adults' experience of stroke: a qualitative review of the literature. *Br. J. Nurs.* 2010;19:241-248. doi: 10.12968/bjon.2010.19.4.46787
38. Australian Institute of Health and Welfare (AIHW). Heart, stroke and vascular diseases—Australian facts 2004. *Cardiovascular Disease Series No 22*. 2004
39. Australian Institute of Health and Welfare (AIHW). Stroke and its management in Australia: an update. *Cardiovascular Disease Series*. 2013:158
40. Australian Stroke Coalition Rehabilitation Working Group. Assessment for rehabilitation: pathway and decision-making tool 2012. 2012;Melbourne: Australian Stroke Coalition
41. Knoflach M, Matosevic B, Rucker M, Furtner M, Mair A, Wille G, Zangerle A, Werner P, Ferrari J, Schmidauer C, Seyfang L, Kiechl S, Willeit J, Austrian Stroke Unit Registry Collaborators. Functional recovery after ischemic stroke-a matter of age: data from the Austrian Stroke Unit Registry. *Neurology*. 2012;78:279-285. doi: 10.1212/WNL.0b013e31824367ab
42. Leys D, Bandu L, Henon H, Lucas C, Mounier-Vehier F, Rondepierre P, Godefroy O. Clinical outcome in 287 consecutive young adults (15 to 45 years) with ischemic stroke. *Neurology*. 2002;59:26-33. doi: 10.1212/wnl.59.1.26
43. Varona JF, Bermejo F, Guerra JM, Molina JA. Long-term prognosis of ischemic stroke in young adults. Study of 272 cases. *J. Neurol.* 2004;251:1507-1514. doi: 10.1007/s00415-004-0583-0
44. Naess H, Waje-Andreassen U, Thomassen L, Nyland H, Myhr KM. Health-related quality of life among young adults with ischemic stroke on long-term follow-up. *Stroke*. 2006;37:1232-1236. doi: 10.1161/01.Str.0000217652.42273.02
45. Stroke Foundation. Clinical Guidelines for Stroke Management. Living Guidelines. https://informme.org.au/en/Guidelines/Clinical-Guidelines-for-Stroke-Management. 2019. Accessed 12 November 2019:Chapter 6.
46. Kersten P, Low JT, Ashburn A, George SL, McLellan DL. The unmet needs of young people who have had a stroke: results of a national UK survey. *Disabil. Rehabil.* 2002;24:860-866. doi: 10.1080/09638280210142167

For peer review only

47. Stroke Foundation. National Stroke Audit. Rehabilitation Services Report 2018. 2018
48. Shipley J, Luker J, Thijs V, Bernhardt J. How can stroke care be improved for younger service users? A qualitative study on the unmet needs of younger adults in inpatient and outpatient stroke care in Australia. *Disabil. Rehabil.* 2019;Jan 7:1-8. doi: https://doi.org/10.1080/09638288.2018.1534278
49. Daniel K, Wolfe CD, Busch MA, McKevitt C. What are the social consequences of stroke for working-aged adults?: A systematic review. *Stroke*. 2009;40:e431-e440. doi: 10.1161/STROKEAHA.108.534487
50. Wei X-J, Liu X-F, Fong KN. Outcomes of return-to-work after stroke rehabilitation: A systematic review. *Br. J. Occup. Ther.* 2016;79:299-308. doi: 10.1177/0308022615624710
51. Glozier N, Hackett ML, Parag V, Anderson CS. The influence of psychiatric morbidity on return to paid work after stroke in younger adults: the Auckland Regional Community Stroke (ARCOS) Study, 2002 to 2003. *Stroke*. 2008;39:1526-1532. doi: 10.1161/strokeaha.107.503219
52. Hackett ML, Glozier N, Jan S, Lindley R. Returning to paid employment after stroke: the Psychosocial Outcomes In StrokE (POISE) cohort study. *PLoS One*. 2012;7:e41795. doi: 10.1371/journal.pone.0041795
53. Baldwin C, Brusco NK. The effect of vocational rehabilitation on return-to-work rates post stroke: a systematic review. *Top. Stroke Rehabil.* 2011;18:562-572. doi: 10.1310/tsr1805-562

For peer review only

Figure Legends

Figure 1 Four post-acute discharge pathways: Inpatient rehabilitation only; inpatient rehabilitation followed by community rehabilitation; home with community rehabilitation; and home with no community rehabilitation

Note. Pathway does not include discharge to supported accommodation.

Figure 2 Rehabilitation service use and outcomes of participants ≤ 65 years in Australasia

Participants in IR at 3 months n=28 (7.7%); at 12months n=3 (<1%)
Participants receiving CR at 3 months n=1 (<1%), at 12 months n=39 (10.7%)
Participants who had not been discharged from their initial acute admission or were still in IR at the time of 3-month follow-up were allocated to the appropriate pathway using CR data collected at 12 months.
LOS (Length of Stay), Age, NIHSS data are median (IQR)
Depression (IDA scale), HRQoL (AQoL-4D), Community ambulator defined as Rivermead Motor Assessment Gross Function >9
12 month outcomes data are % of group, except Return to Work data which are % of those working prior to stroke
Missing 12 month outcomes data: Unknown Missing n=10/69 (14.5%); Alive Missing n=59/69 (85.5%)

Figure 3 Rehabilitation service use and outcomes of participants ≤ 65 years in United Kingdom

Participants in inpatient rehabilitation at 3 months n=8 (5.0%); at 12 months n=1 (<1%)
Participants receiving community rehabilitation services at 3months n=1 (<1%), at 12 months n=11 (6.8%)
Participants who had not been discharged from their initial acute admission or were still in IR at the time of 3 month follow-up were allocated to the appropriate pathway using CR data collected at 12 months.
LOS (Length of Stay), Age, NIHSS data are median (IQR)
Depression (IDA scale), HRQoL (AQoL-4D), Community ambulator defined as Rivermead Motor Assessment Gross Function >9
12 month data are % of group, except Return to Work data which are % of participants in group working prior to stroke
Missing 12 month outcomes data: Unknown Missing n=15/71 (21.1%); Alive Missing n=56/71 (78.9%)

Figure 4 Rehabilitation service use and outcomes of participants ≤ 65 years in South East Asia

Participants in inpatient rehabilitation at 3 months n=0; at 12 months n=0
Participants receiving community rehabilitation services at 3 months n=2 (1.4%), at 12 months n=0
Participants who had not been discharged from their initial acute admission or were still in IR at the time of 3 month follow-up were allocated to the appropriate pathway using CR data collected at 12 months.
LOS (Length of Stay), Age, NIHSS data are median (IQR)
Depression (IDA scale), HRQoL (AQoL-4D), Community ambulator defined as Rivermead Gross Motor Function >9
12 month data are % of group, except Return to Work data which are % of participants in group working prior to stroke
Missing 12 month outcomes data: Unknown Missing n=6/40 (15%); Alive Missing n=34/40 (85%)

For peer review only

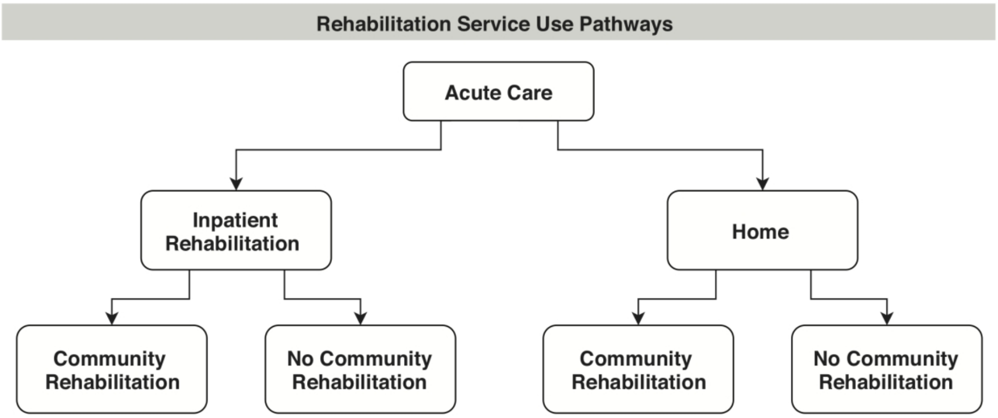

Figure 1 Four post-acute discharge pathways: Inpatient rehabilitation only; inpatient rehabilitation followed by community rehabilitation; home with community rehabilitation; and home with no community rehabilitation Note. Pathway does not include discharge to supported accommodation.

173x80mm (300 x 300 DPI)

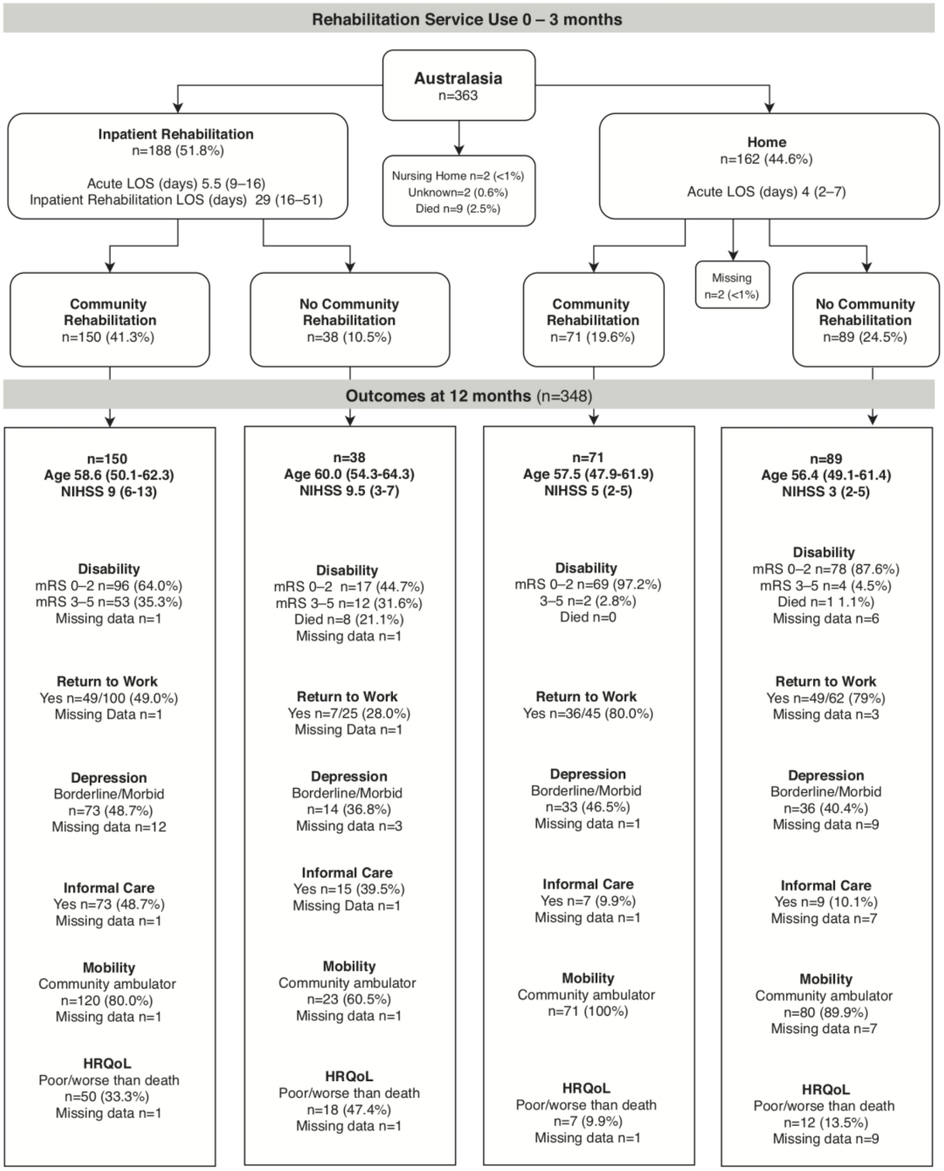

Figure 2 Rehabilitation service use and outcomes of participants ≤ 65 years in AustralasiaParticipants in IR at 3 months n=28 (7.7%); at 12months n=3 (<1%)Participants receiving CR at 3 months n=1 (<1%), at 12 months n=39 (10.7%)Participants who had not been discharged from their initial acute admission or were still in IR at the time of 3-month follow-up were allocated to the appropriate pathway using CR data collected at 12 months.LOS (Length of Stay), Age, NIHSS data are median (IQR)Depression (IDA scale), HRQoL (AQoL-4D), Community ambulator defined as Rivermead Motor Assessment Gross Function >912 month outcomes data are % of group, except Return to Work data which are % of those working prior to strokeMissing 12 month outcomes data: Unknown Missing n=10/69 (14.5%); Alive Missing n=59/69 (85.5%)

173x214mm (300 x 300 DPI)

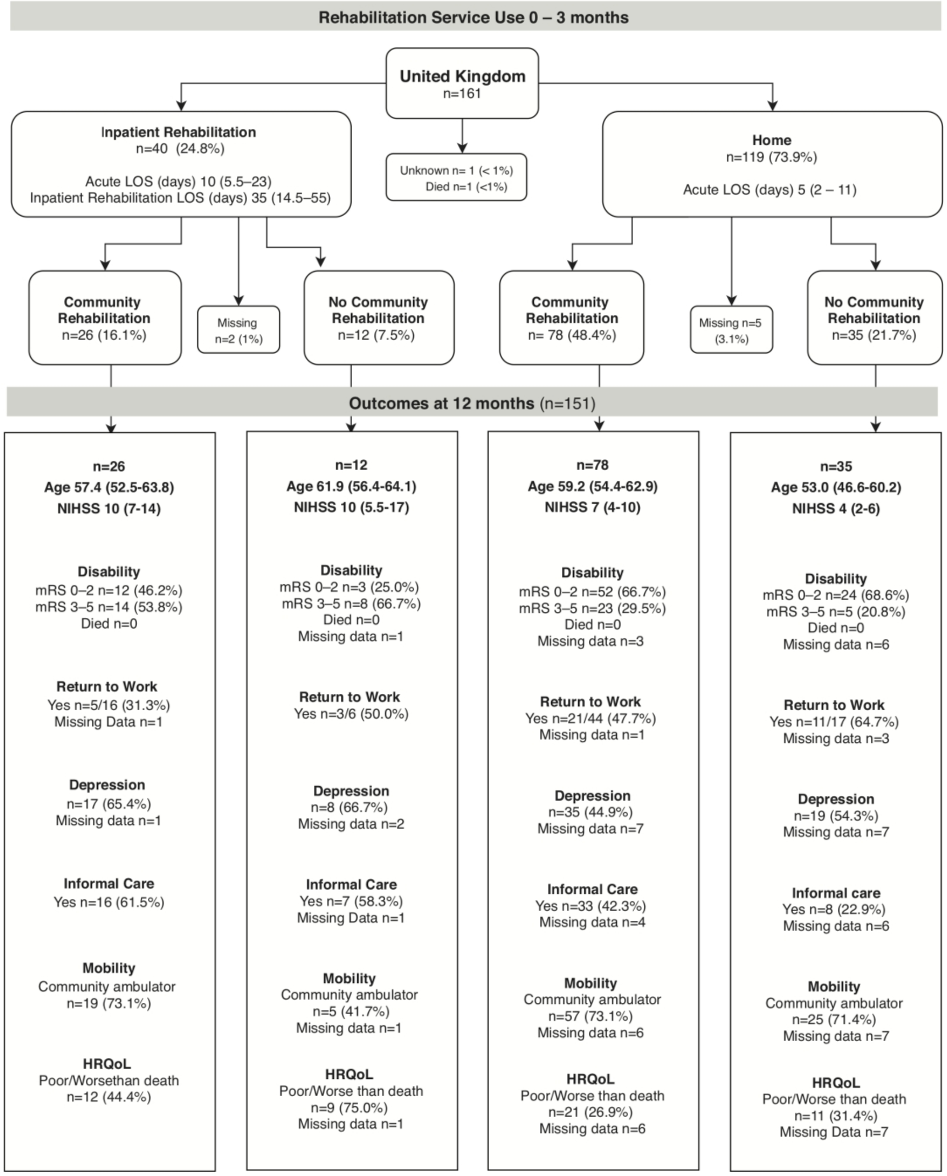

Figure 3 Rehabilitation service use and outcomes of participants ≤ 65 years in United Kingdom
Participants in inpatient rehabilitation at 3 months n=8 (5.0%); at 12 months n=1 (<1%)
Participants receiving community rehabilitation services at 3months n=1 (<1%), at 12 months n=11 (6.8%)
Participants who had not been discharged from their initial acute admission or were still in IR at the time of 3 month follow-up were allocated to the appropriate pathway using CR data collected at 12 months.
LOS (Length of Stay), Age, NIHSS data are median (IQR)
Depression (IDA scale), HRQoL (AQoL-4D), Community ambulator defined as Rivermead Motor Assessment Gross Function >9
12 month data are % of group, except Return to Work data which are % of participants in group working prior to stroke
Missing 12 month outcomes data: Unknown Missing n=15/71 (21.1%); Alive Missing n=56/71 (78.9%)

173x214mm (300 x 300 DPI)

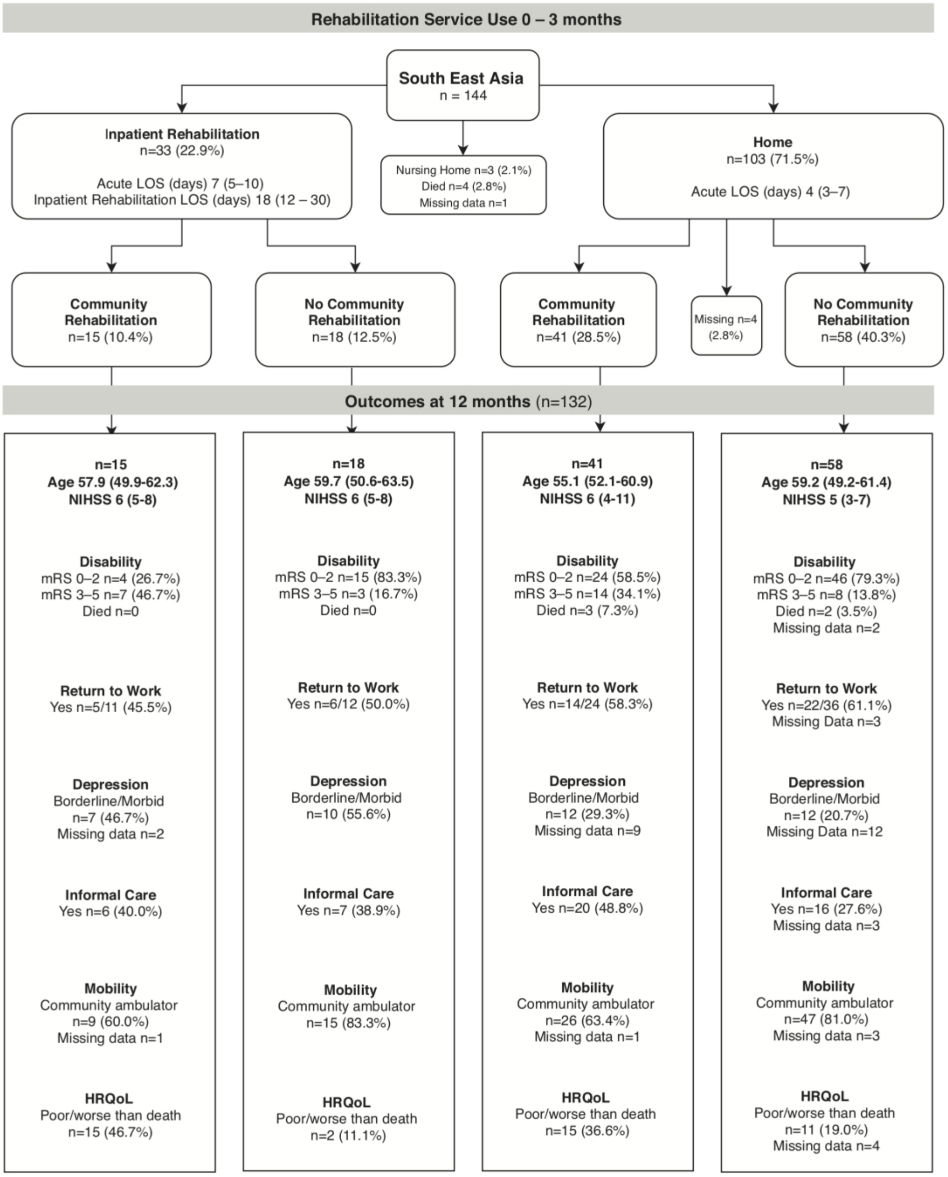

Figure 4 Rehabilitation service use and outcomes of participants ≤ 65 years in South East Asia
Participants in inpatient rehabilitation at 3 months n=0; at 12 months n=0
Participants receiving community rehabilitation services at 3 months n=2 (1.4%), at 12 months n=0
Participants who had not been discharged from their initial acute admission or were still in IR at the time of 3 month follow-up were allocated to the appropriate pathway using CR data collected at 12 months.
LOS (Length of Stay), Age, NIHSS data are median (IQR)
Depression (IDA scale), HRQoL (AQoL-4D), Community ambulator defined as Rivermead Gross Motor Function >9
12 month data are % of group, except Return to Work data which are % of participants in group working prior to stroke
Missing 12 month outcomes data: Unknown Missing n=6/40 (15%); Alive Missing n=34/40 (85%)

173x214mm (300 x 300 DPI)

Supplement 1

S-Table 1 Proportion of stroke survivors using different rehabilitation services in each region by two major prognostic factors: stroke severity and age

Region	Stroke Severity & Age	n	Inpatient Rehabilitation & Community Rehabilitation	Inpatient Rehabilitation & No Community Rehabilitation	Home & Community Rehabilitation	Home & No Rehabilitation Service Use
			n=150	n=38	n=71	n=89
AUS	Mild	209	59 (28%)	12 (6%)	58 (28%)	80 (38%)
	Mod	103	67 (65%)	15 (15%)	12 (12%)	9 (9%)
	Severe	36	24 (67%)	11 (31%)	1 (3%)	0 (0%)
	18-45	54	23 (43%)	1 (2%)	14 (26%)	16 (30%)
	46-65	294	127 (43%)	37 (13%)	57 (19%)	73 (25%)
			n=26	n=12	n=78	n=35
UK	Mild	87	8 (9%)	4 (5%)	45 (52%)	30 (34%)
	Mod	53	15 (28%)	5 (9%)	28 (53%)	5 (9%)
	Severe	11	3 (27%)	3 (27%)	5 (45%)	0 (0%)
	16-45	17	2 (12%)	0 (0%)	7 (41%)	8 (47%)
	46-65	134	24 (18%)	12 (9%)	71 (53%)	27 (20%)
			n=15	n=18	n=41	n=58
SE Asia	Mild	93	11 (12%)	12 (13%)	24 (26%)	46 (49%)
	Mod	34	4 (12%)	6 (18%)	14 (41%)	10 (29%)
	Severe	5	0 (0%)	0 (0%)	3 (60%)	2 (40%)
	16-45	21	2 (10%)	3 (14%)	5 (24%)	11 (52%)
	46-65	111	13 (12%)	15 (14%)	36 (32%)	47 (42%)

Note. AUS=Australia, UK=United Kingdom, SE Asia=South East Asia, Mod=Moderate

For peer review only

Supplement 2

S-Table 2. Recommendations for post stroke rehabilitation from Australia, UK and Singapore clinical guidelines during AVERT

Guideline	Recommendation
Australia (NSF, 2005 & 2007)[35,36]	All people admitted to hospital with stroke and who require rehabilitation should be treated in a comprehensive or rehabilitation stroke unit with an interdisciplinary team (Level I)<br><br>If ongoing inpatient rehabilitation is needed, care should be provided in either a stroke rehabilitation unit or a general rehabilitation unit (Grade A; Level I)<br><br>Rehabilitation in the community is equally effective if delivered in the hospital via outpatients, or day hospital, or in the community, and should be offered to all stroke patients as needed (Grade A; Level I)<br><br>Where comprehensive interdisciplinary community rehabilitation services and carer support services are available, early supported discharge services may be provided for people with mild to moderate disability (Level I)
UK (RCP 3rd edition, 2008)[34]	Once a patient is medically investigated, treated and stable, rehabilitation should be delivered by a specialist stroke service (in an inpatient unit, or a day-hospital unit, or at home)<br><br>All patients discharged home directly after acute treatment but with residual problems should be followed up by specialist stroke rehabilitation services<br><br>Domiciliary rehabilitation services should be commissioned as part of an 'early supported discharge' scheme to deliver specialist rehabilitation at home in liaison with inpatient services, as well as in the long-term<br><br>Patients should only be discharged early (before the end of acute rehabilitation) from National Clinical Guideline for Stroke hospital if there is a specialist stroke rehabilitation team able to continue rehabilitation in the community from the day of transfer and if the patient is able to transfer safely from bed to chair, and if other problems can be safely managed at home
Singapore (MoH, 2009)[31]	Stroke patients should receive organized inpatient multidisciplinary rehabilitation (Grade A, Level 1+)<br><br>If able to do so, stroke patients should be encouraged to participate in more intensive rehabilitation particularly in the first six months (Grade A, Level 1+)

Levels of evidence
NSF (2005, 2007)[35,36]
Level I Evidence obtained from a systematic review of all relevant randomised controlled trials. Grade A recommendation: A Body of evidence can be trusted to guide practice

Singapore MoH (2009)[31]
Level 1+: Well conducted meta-analyses, systematic reviews of RCTs, or RCTs with a low risk of bias.
Grade A Recommendation: At least one meta-analysis, systematic review of RCTs, or RCT rated as 1+ + and directly applicable to the target population; or A body of evidence consisting principally of studies rated as 1+, directly applicable to the target population, and demonstrating overall consistency of results

For peer review only

STROBE Statement—Checklist of items that should be included in reports of ***cohort studies***

	Item No	Recommendation	Page No
**Title and abstract**	1	(*a*) Indicate the study's design with a commonly used term in the title or the abstract	1
		(*b*) Provide in the abstract an informative and balanced summary of what was done and what was found	2
**Introduction**			
Background/rationale	2	Explain the scientific background and rationale for the investigation being reported	5-6
Objectives	3	State specific objectives, including any prespecified hypotheses	6
**Methods**			
Study design	4	Present key elements of study design early in the paper	7
Setting	5	Describe the setting, locations, and relevant dates, including periods of recruitment, exposure, follow-up, and data collection	7
Participants	6	(*a*) Give the eligibility criteria, and the sources and methods of selection of participants. Describe methods of follow-up	7-8
		(*b*) For matched studies, give matching criteria and number of exposed and unexposed	N/A
Variables	7	Clearly define all outcomes, exposures, predictors, potential confounders, and effect modifiers. Give diagnostic criteria, if applicable	8-9
Data sources/ measurement	8*	For each variable of interest, give sources of data and details of methods of assessment (measurement). Describe comparability of assessment methods if there is more than one group	8-9
Bias	9	Describe any efforts to address potential sources of bias	9
Study size	10	Explain how the study size was arrived at	7
Quantitative variables	11	Explain how quantitative variables were handled in the analyses. If applicable, describe which groupings were chosen and why	9
Statistical methods	12	(*a*) Describe all statistical methods, including those used to control for confounding	9
		(*b*) Describe any methods used to examine subgroups and interactions	8
		(*c*) Explain how missing data were addressed	9
		(*d*) If applicable, explain how loss to follow-up was addressed	N/A
		(*e*) Describe any sensitivity analyses	N/A
**Results**			
Participants	13*	(a) Report numbers of individuals at each stage of study—eg numbers potentially eligible, examined for eligibility, confirmed eligible, included in the study, completing follow-up, and analysed	9-10
		(b) Give reasons for non-participation at each stage	8-13
		(c) Consider use of a flow diagram	Figures 1-4
Descriptive data	14*	(a) Give characteristics of study participants (eg demographic, clinical, social) and information on exposures and potential confounders	10-11
		(b) Indicate number of participants with missing data for each variable of interest	8-13, Table 1, Figures 2,3,4
		(c) Summarise follow-up time (eg, average and total amount)	

For peer review only - http://bmjopen.bmj.com/site/about/guidelines.xhtml

Outcome data	15*	Report numbers of outcome events or summary measures over time	12-13, Figures 2,3,4

For peer review only - http://bmjopen.bmj.com/site/about/guidelines.xhtml
For peer review only

Main results	16	(*a*) Give unadjusted estimates and, if applicable, confounder-adjusted estimates and their precision (eg, 95% confidence interval). Make clear which confounders were adjusted for and why they were included	N/A
		(*b*) Report category boundaries when continuous variables were categorized	10-11
		(*c*) If relevant, consider translating estimates of relative risk into absolute risk for a meaningful time period	N/A
Other analyses	17	Report other analyses done—eg analyses of subgroups and interactions, and sensitivity analyses	
**Discussion**			
Key results	18	Summarise key results with reference to study objectives	13-16
Limitations	19	Discuss limitations of the study, taking into account sources of potential bias or imprecision. Discuss both direction and magnitude of any potential bias	16-17
Interpretation	20	Give a cautious overall interpretation of results considering objectives, limitations, multiplicity of analyses, results from similar studies, and other relevant evidence	13-17
Generalisability	21	Discuss the generalisability (external validity) of the study results	16-17
**Other information**			
Funding	22	Give the source of funding and the role of the funders for the present study and, if applicable, for the original study on which the present article is based	18

*Give information separately for exposed and unexposed groups.

Note: An Explanation and Elaboration article discusses each checklist item and gives methodological background and published examples of transparent reporting. The STROBE checklist is best used in conjunction with this article (freely available on the Web sites of PLoS Medicine at http://www.plosmedicine.org/, Annals of Internal Medicine at http://www.annals.org/, and Epidemiology at http://www.epidem.com/). Information on the STROBE Initiative is available at http://www.strobe-statement.org.

For peer review only

For peer review only - http://bmjopen.bmj.com/site/about/guidelines.xhtml
